# Expanding the eggshell colour gamut: uroerythrin and bilirubin from tinamou (*Tinamidae*) eggshells

**DOI:** 10.1038/s41598-020-68070-7

**Published:** 2020-07-09

**Authors:** Randy Hamchand, Daniel Hanley, Richard O. Prum, Christian Brückner

**Affiliations:** 1 Department of Chemistry, University of Connecticut, Unit 3060, Storrs, CT 06269-3060 USA; 2 Department of Biology and Environmental Sciences, Long Island University - Post, Brookville, NY 11548 USA; 3 Department of Ecology and Evolutionary Biology, Peabody Museum of Natural History, Yale University, New Haven, CT 06520-8105 USA

**Keywords:** Biosynthesis, Animal physiology, Chemical modification, Structure elucidation

## Abstract

To date, only two pigments have been identified in avian eggshells: rusty-brown protoporphyrin IX and blue-green biliverdin IXα. Most avian eggshell colours can be produced by a mixture of these two tetrapyrrolic pigments. However, tinamou (*Tinamidae*) eggshells display colours not easily rationalised by combination of these two pigments alone, suggesting the presence of other pigments. Here, through extraction, derivatization, spectroscopy, chromatography, and mass spectrometry, we identify two novel eggshell pigments: yellow–brown tetrapyrrolic bilirubin from the guacamole-green eggshells of *Eudromia elegans,* and red–orange tripyrrolic uroerythrin from the purplish-brown eggshells of *Nothura maculosa*. Both pigments are known porphyrin catabolites and are found in the eggshells in conjunction with biliverdin IXα. A colour mixing model using the new pigments and biliverdin reproduces the respective eggshell colours. These discoveries expand our understanding of how eggshell colour diversity is achieved. We suggest that the ability of these pigments to photo-degrade may have an adaptive value for the tinamous.

## Introduction

**Figure 1:**
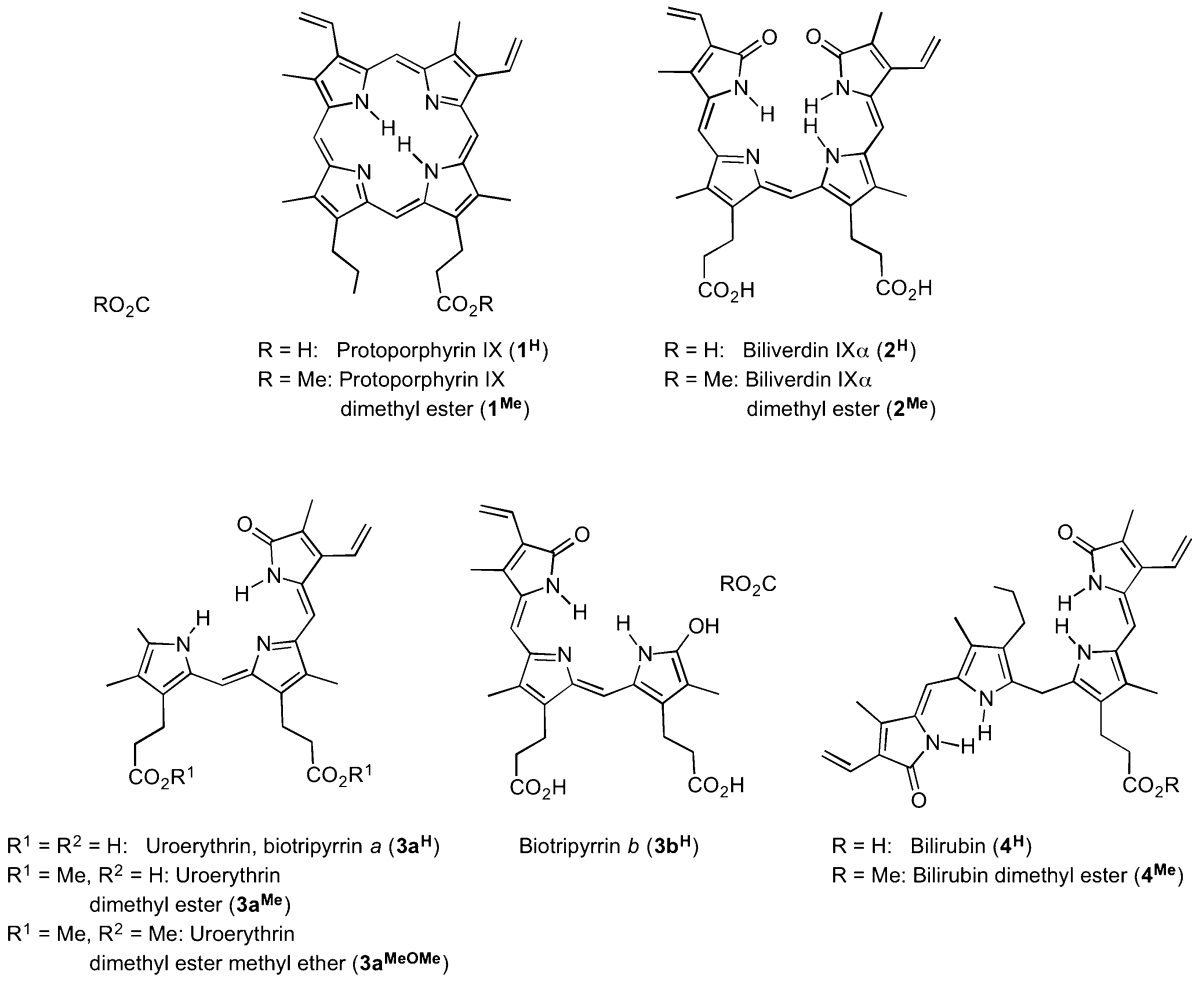
Molecular structures of the two eggshell pigments (**1**^**H**^ and **2**^**H**^) identified to date, and the two new pigments (**3a**^**H**^/**3b**^**H**^ and **4**^**H**^) reported here, isolated as their dimethyl esters (**3**^**Me**^ and **4**^**Me**^, respectively); structure of the literature-known uroerythrin dimethyl ester methyl ether **3a**^**MeOMe**^ is also included.

Birds’ eggs are found in an expansive variety of shapes, sizes, and colourings^1^. The diverse array of appearances found across *Aves* is achieved—in large part—through a combination of structural features, solid or patterned colorations, the use of two different dyes, and differential pigment deposition. Eggshell pigments are embedded within the white calcium carbonate matrix of the egg and within a thin outer proteinaceous layer called the cuticle^2–4^. These pigments are believed to play a key role in crypsis^5,6^, although other, possibly dynamic^7,8^, roles in inter- and intra-species signalling^5,9–12^ are also possible. In addition, these pigments may provide a range of structural, thermoregulatory, UV-protective, and photo-dependent antimicrobial benefits^5,13–18^. Despite the diversity of observable colours, there is a universal consensus that all of these colors are generated by only two pigments^1,4,19–22^: the tetrapyrrolic compounds protoporphyrin IX (referred to here as **1**^**H**^, rusty-brown) and biliverdin IXα (**2**^**H**^, blue-green) (Fig. 1).

**Figure 2:**
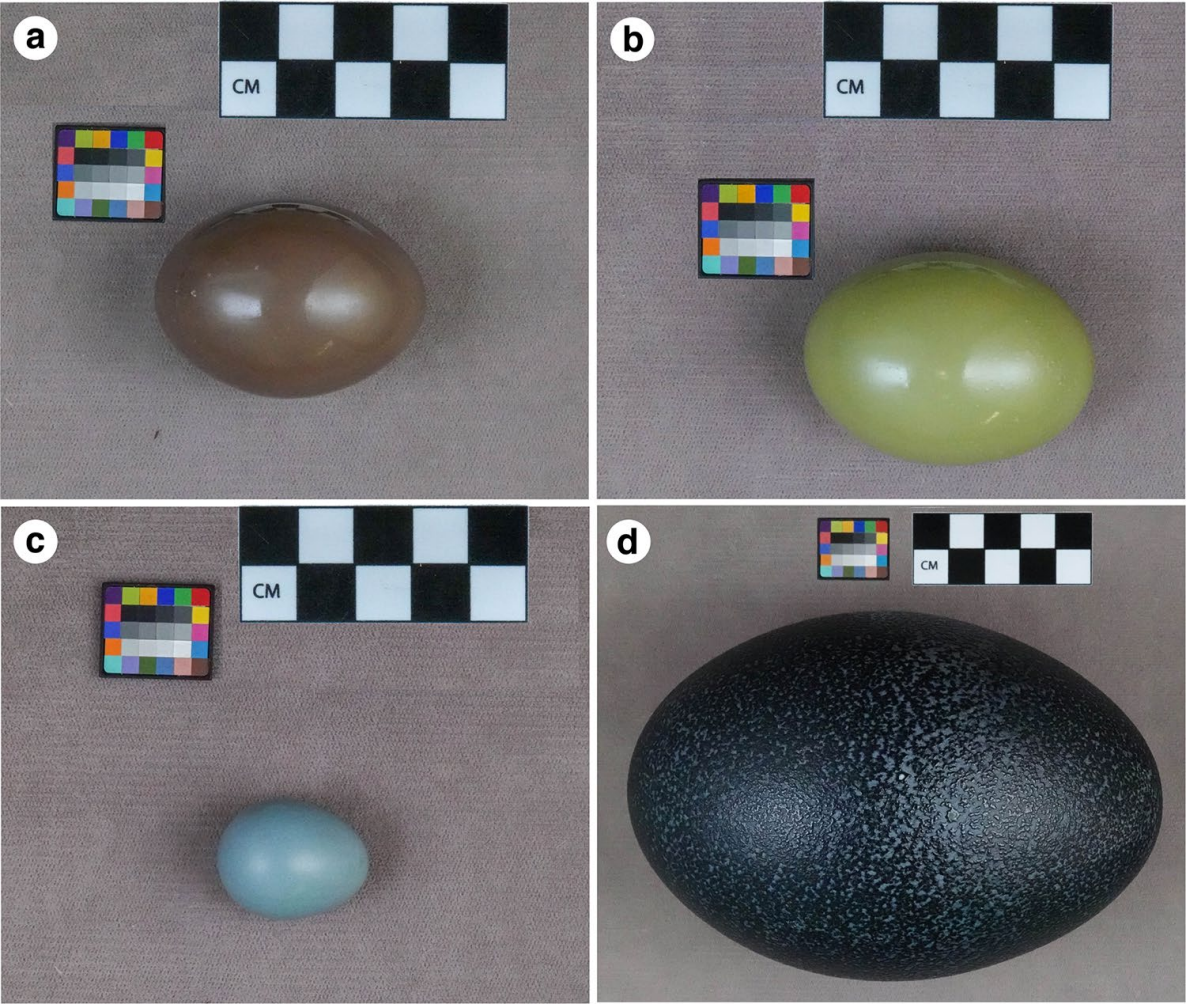
Photographs of eggshells of (**a**) *Nothura maculosa,* (**b**) *Eudromia elegans*, (**c**) *Turdus migratorius*, and (**d**) *Dromaius novaehollandiae*. Image credits: Richard O. Prum.

Tinamous (*Tinamidae*) are ground-dwelling, chicken-like, flying paleognathes native to Central and South America^23^. Tinamou eggs exhibit a diversity of bright colours, ranging from blues and greens to exotic greys and deep purplish-browns. Their eggshell surfaces have a distinctive glossy, porcelain-like appearance generated by nanostructured surface calcite and calcium phosphate crystals^3^. Interestingly, only the blue-green pigment biliverdin has been previously detected in the purplish-brown eggshells of the Spotted Nothura (*Nothura maculosa*; Fig. 2a) and the guacamole-green eggshell of the Elegant Crested Tinamou (*Eudromia elegans*; Fig. 2b)^20,24^. However, these tinamou eggshells differ strikingly in colour from other bird eggshells, including the biliverdin-only blue-green eggshells of the American robin (*Turdus migratorius*; Fig. 2c) and the dark-green eggshells of the emu (*Dromaius novaehollandiae*; Fig. 2d)^1^. We thus hypothesized that the purplish and green hues of the *N. maculosa* and *E. elegans* eggshells*,* respectively, are generated by mixing biliverdin with other, yet unknown, pigments. Therefore, we re-examined these tinamou eggshells, specifically extracting and identifying their pigments, and analysed their contributions to the observed eggshell coloration.

## Results

### *Nothura maculosa* eggshell extraction

**Figure 3:**
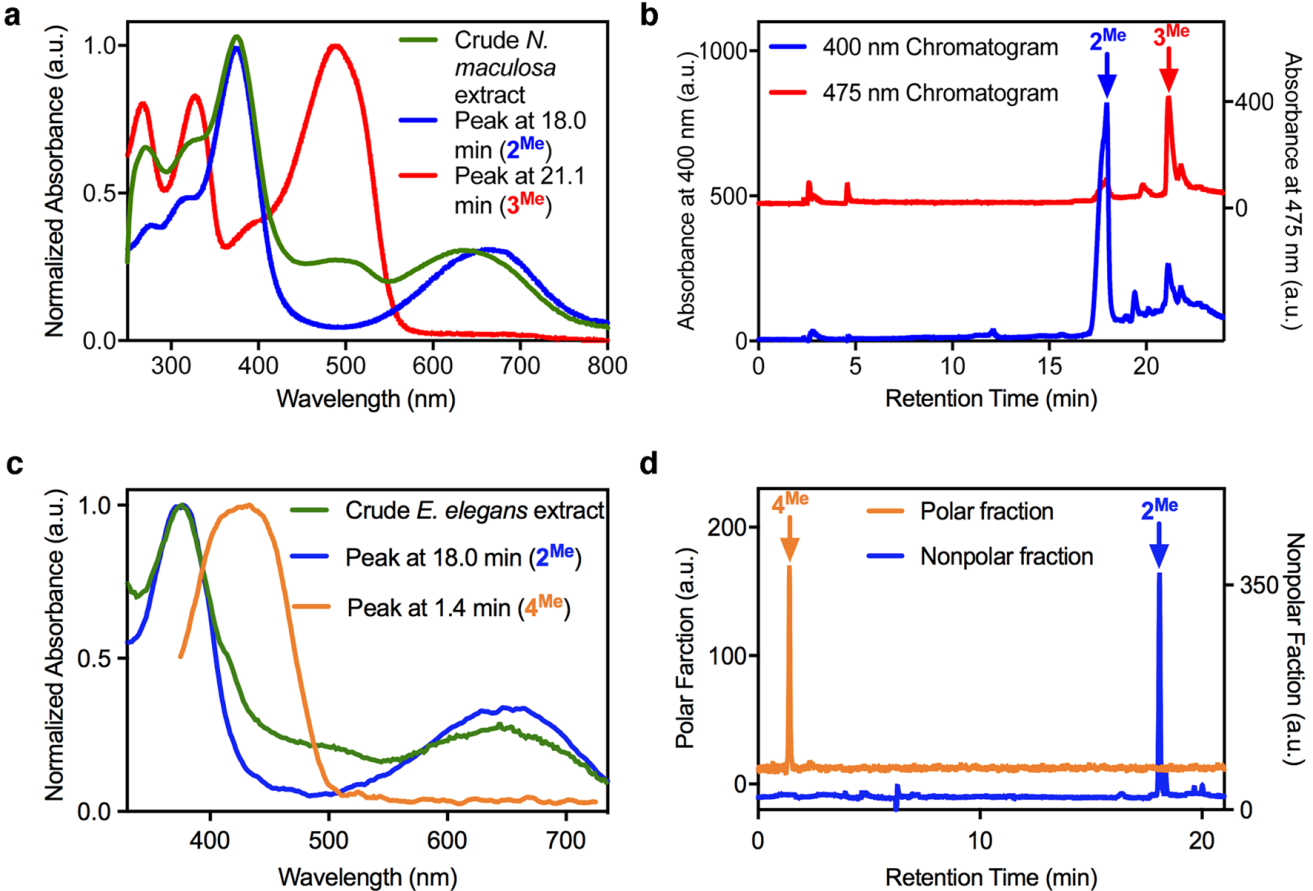
(**a**) Normalized UV–Vis spectra of the raw *N. maculosa* eggshell extract (green trace, EtOAc), extracted biliverdin **2**^**Me**^ (blue trace, MeOH), and extracted uroerythrin **3**^**Me**^ (red trace, MeOH). (**b**) HPLC traces of the raw extract from purple *N. maculosa* eggshells at two different wavelengths of detection. (**c**) Normalized UV–Vis spectra of the raw *E. elegans* eggshell extract (green trace, EtOAc), and of the two major components: biliverdin **2**^**Me**^ (blue trace, in MeOH) and bilirubin **4**^**Me**^ (orange trace, in MeOH). (**d**) NP-HPLC traces (at 400 nm detection wavelength) of the polar and nonpolar fractions from the *E. elegans* extract.

We applied a variation of a classic methanolic sulfuric acid-based eggshell pigment extraction protocol that esterifies the carboxylic acids of the pigments, yielding efficient partitioning of the pigments into an organic phase^25–28^. The UV–Vis absorption spectrum of the raw organic extract from the purplish-brown *N. maculosa* eggshell exhibits characteristic features of a bilin-type spectrum (i.e. two broad bands near 380 nm and 650 nm)^29,30^, as well as prominent bands centred at 325 and ~ 490 nm (Fig. 3a, green). The absence of any sharp feature in the UV–Vis absorption spectrum near 400 nm arising from the diagnostic Soret band of porphyrins suggests none are present.

Analysis of the crude extract using normal-phase high-performance liquid chromatography (NP-HPLC) revealed the presence of two major chromophores and some minor components (Fig. 3b). The first component, eluting at 18.0 min and absorbing strongly at 400 nm (Fig. 3b, blue), displayed a typical biliverdin UV–Vis spectrum^29,30^ (Fig. 3a, blue). The compound proved to be identical in retention time, composition (C_35_H_39_N_4_O_6_ for [M + H]^+^ as per electrospray high-resolution mass spectrometry in the positive ion mode (ESI + HR-MS)), and molecular ion fragmentation pattern to biliverdin dimethyl ester **2**^**Me**^ extracted from emu (*Dromaius novaehollandiae*) eggshells^28^ and of commercial samples (Supplementary Figs. S1–4).

Inspecting the 475 nm detection chromatogram (Fig. 3b, red), we observe that the biliverdin peak (at 18.0 min) is, as expected, diminished; however, a second major peak, eluting at 21.1 min, is prominent. This peak is associated with a pigment that appears red–orange to the human eye and that possesses a three-band UV–Vis spectrum with features centred at 269, 325, and 495 nm (Fig. 3a, red). When compared to bilins, such blue-shifted spectra correlate with those of conjugated tripyrrolic compounds^30,31^. The pigment’s composition was determined to be C_27_H_31_N_3_O_6_, (for [M + H]^+^ as per ESI + HR-MS; Supplementary Fig. S5), and tandem HR-MS^2^ experiments supported the presence of a tripyrrolic pigment (Supplementary Fig. S6). Taken together, the data identify the orange pigment as the dimethyl ester **3**^**Me**^ of uroerythrin **3a**^**H**^^32^ (for a discussion of the presence of its isomer **3b**^**H**^, see below). The UV–Vis absorption spectrum of the crude extract allowed us to estimate the relative molar ratio of the two pigments biliverdin **2**^**Me**^ and uroerythrin **3**^**Me**^ to be about 3.5:1, with biliverdin **2**^**Me**^ being present in the range of 20–40 nmol g^−1^ eggshell. The colour mixing model described below provides an independently verified match of the pigment ratios.

We experimentally corroborated with independently sourced protoporphyrin **1**^**H**^ and biliverdin **2**^**H**^ that our pigment extraction protocol did not lead to their degradation, in general, or to the production of uroerythrin **3**^**Me**^, in particular. Inversely, we found that purified uroerythrin extracts degraded in solution or as a film within days at ambient conditions (air, room temperature, light), but also—albeit slower—at − 20 °C in the dark. This speaks of the general lability of the pigment—an aspect of possible biological function, see below—and the need for rapid analysis after extraction for accurate quantitation.

**Figure 4:**
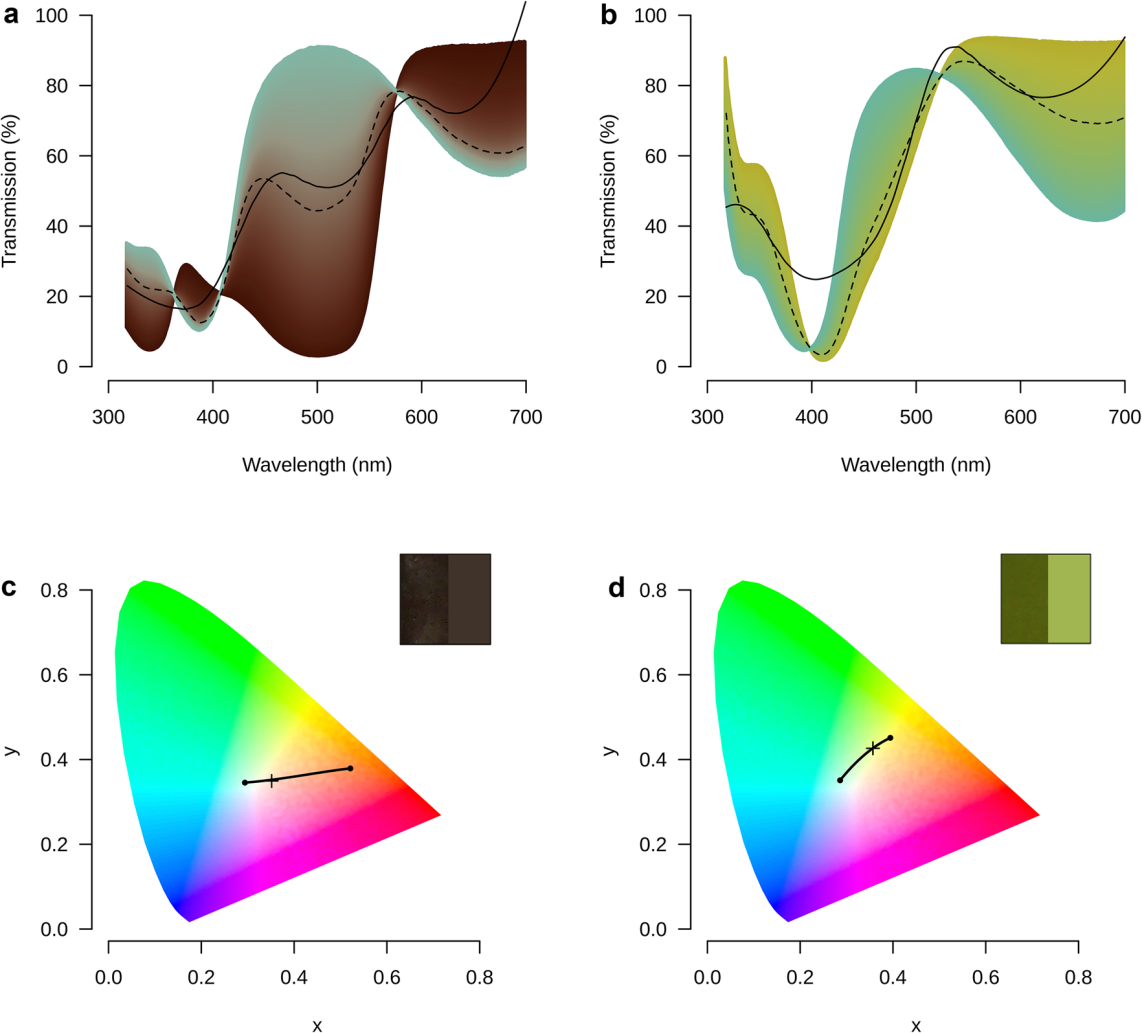
Transmission spectra for the variable subtractive mixtures of (**a**) biliverdin and uroerythrin from *N. maculosa* and (**c**) biliverdin and bilirubin from *E. elegans* eggshells, respectively plotted in their modelled colours. Measured spectral reflectance of those eggshells (solid) is compared to the best predicted spectrum (dashed). All spectra are depicted from 318 to 700 nm. The colour of each spectrum is calculated from its spectrum, and the brightness of those colours was modified to approximate the reflectance of whole eggshells (solid black lines). Intermediate spectra for (**b**) biliverdin and uroerythrin and (**d**) biliverdin and bilirubin were plotted within the CIE coordinate space (small black dots) varying from entirely biliverdin (left most large dot) to entirely the novel pigment (rightmost large dot). For comparison, the reflectance values of whole eggshells are plotted (+ symbol) within each coordinate space. Insets (**b**) and (**d**): Colour swatch for our best prediction (left) compared against a close-up photograph of the surface colour (right).

Extractions of *N. maculosa* eggshells using EDTA, followed by ESI + MS–MS analysis of the extracts separated by reverse-phase HPLC (RP-HPLC), confirmed that the pigments were, as expected, present in the eggshells in their diacid forms **2**^**H**^ and **3**^**H**^ (Supplementary Figs. S7–9, S12). Furthermore, this analysis also shed more light on the nature of the minor pigments present, one of which could be identified as a uroerythrin isomer^33^ (Supplementary Fig. S7) and one to be likely a formyldipyrrinone^34^ (Supplementary Fig. S13). Of note, trace amounts of bilirubin **4**^**H**^ were detectable in the EDTA extracts through ESI + HR-MS, also (Supplementary Figs. S10–11). However, the absence of a **4**^**H**^ chromophore in the 400 nm RP-HPLC chromatogram (Supplementary Fig. S7), coupled with our colour blending model discussed below (cf. Figure 4), suggests that bilirubin **4**^**H**^ does not play any noticeable role in colouring *N. maculosa* eggshells.

### *Eudromia elegans* eggshell extraction

The guacamole-green eggshells of *E. elegans* were also extracted and analysed using NP-HPLC, UV–Vis spectroscopy, and ESI + HR-MS spectrometry as described above. The UV–Vis spectrum of the raw extract is overall bilin-like (i.e. broad bands near 380 nm and 650 nm), with the exception of the presence of a small shoulder at ~ 420 nm and a stronger than expected absorption in the range between ~ 450 and 550 nm (Fig. 3c, green). The lack of a sharp Soret band near 400 nm, again, indicates that porphyrins are not present. The crude sample was fractionated into a non-polar (eluent ethyl acetate), blue-green fraction from a more polar (eluent 4:1 ethyl acetate:methanol) yellow–brown-coloured fraction.

HPLC analysis showed that the non-polar fraction contains a single major chromophore (Fig. 3d, blue), identified as biliverdin dimethyl ester **2**^**Me**^ (Supplementary Fig. S16). Likewise, the polar fraction also contains only one major chromophore (Fig. 3d, orange). The composition of this pigment (C_35_H_40_N_4_O_6_Na as per ESI + HR-MS for its sodium adduct [M + Na]^+^) and diagnostic UV–Vis spectrum (single band absorption spectrum centred at ~ 440 nm; Fig. 3c, orange) characterize it as bilirubin dimethyl ester **4**^**Me**^^29^. Accordingly, it also proved to be identical in retention time, UV–Vis absorbance profile, and gas phase behaviour under ESI + MS conditions to independently sourced bilirubin dimethyl ester (Supplementary Figs. S15 and S17–18). The presence of the diacid forms of **2**^**H**^ and **4**^**H**^ in the eggshells was also confirmed using EDTA eggshell extractions, followed by RP-HPLC and ESI + MS–MS analysis of the extracts (Supplementary Figs. S19, S21 and S23).

An analysis of the UV–Vis spectrum of the crude yellow extract approximates the molar ratio of **4**^**Me**^:**2**^**Me**^ to be roughly 0.2:1, with biliverdin **2**^**Me**^ present in the range of 5–10 nmol g^−1^ eggshell. Bilirubin **4**^**Me**^ proved to be quite unstable under the extraction conditions and in the crude extract solutions; handling of bilirubin samples for even short periods under ambient conditions (including light exposure) resulted in decomposition and formation of, inter alia, the oxidation product, biliverdin, resulting in an underestimation of the bilirubin contents. Accordingly, the eggshell colour mixing model described below also suggests that the molar ratio of bilirubin:biliverdin in the native eggshells is significantly higher than we estimated based on the optical data of the crude extract.

### Colour mixing models

Employing colour mixing models^35^, we find that combinations of biliverdin **2**^**Me**^ with uroerythrin **3**^**Me**^ or bilirubin **4**^**Me**^ could generate approximate colour matches (within 0.01 and 0.1 just noticeable difference, respectively) to the reflectance spectra of the surfaces of the *N. maculosa* and *E. elegans* eggshells, respectively. The predicted reflectance spectra closely approximated the measured reflectance spectra (Fig. 4a, b), and the modelled colours were very similar in appearance (Fig. 4c, d). These findings support the conclusion that the eggshell colours of both species are generated by the previously identified biliverdin pigment in combination with the previously unknown colorants: uroerythrin for *N. maculosa* and bilirubin for *E. elegans.* Notably, the purplish-brown colour of the *N. maculosa* eggshells did not require the presence of a brown porphyrin.

Additionally, these models predicted a ratio of biliverdin to uroerythrin of 3.74:1, which closely (within 7%) approximated the relative molar ratio of these pigments estimated by UV–Vis spectroscopy of the crude extract (see above). By contrast, the colour mixing model predicted a bilirubin to biliverdin molar ratio of 1.77:1, which is much larger than the spectroscopically estimated ratio of 0.2:1 (see above). This discrepancy is rationalized by the decomposition (oxidation) of the bilirubin and conversion to biliverdin under the extraction conditions. Additionally, the shape of the spectral reflectance curves for both eggshell surfaces (Fig. 4a, b, solid line) were closely approximated by those predicted by these unique admixtures of biliverdin and novel colourants (Fig. 4a, b, dashed line).

## Discussion

Contrary to previous reports that found only biliverdin in *N. maculosa* and *E. elegans* eggshells^20,24^, this study also discovered the orange pigment uroerythrin **3**^**H**^ and the yellow–brown pigment bilirubin **4**^**H**^, respectively. We can confidently conclude that both of these newly found pigments are genuine eggshell pigments as we experimentally verified that they are not artefacts generated in the extraction process. Additionally, these results are supported by colour mixing models which found that unique combinations of these pigments would generate the unusual surface colours of these eggshells. Furthermore, the excellent colour matches generated by our colour mixing models suggest that the presence of other minor pigments, such as the light-yellow dipyrrolic degradation products seen in the extract of the *N. maculosa* eggshells, do not contribute to the colour of the eggshells.

The orange-red tripyrrolic pigment uroerythrin **3**^**H**^ is a member of the urochromes, pigments arising from haem catabolism and present in, for example, human urine^32^. Uroerythrin is excreted in particularly high levels in individuals exhibiting metabolic pathologies and is associated with increased stress levels^32^. The biosynthesis of uroerythrin is not well understood; however, its origin as an oxidative degradation product of biliverdin **2**^**H**^ is assumed^32^. Model studies also hint at its chemical sensitivity^36^. Biliverdin, as well as bilirubin, were shown to be able to photosensitize oxygen leading to their own photodegradation^33,37^.

**Figure 5:**
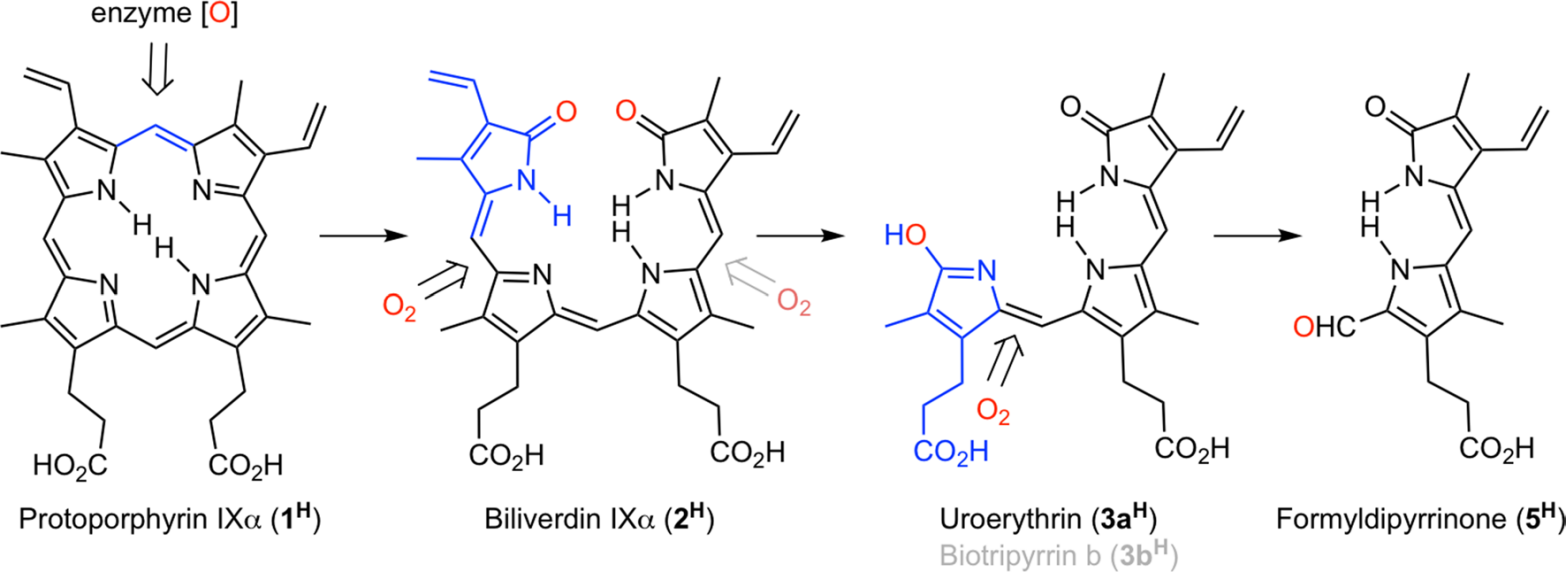
Degradation chain of protoporphyrin IX via biliverdin **2H** to form uroerythrin **3a**^**H**^ (biotripyrrin b) and formydipyrrinone **5**^**H**^. The pyrrolic fragments lost in the subsequent products are indicated in blue, the oxygens introduced in the oxidation step in red.

In the HPLC trace of the crude EDTA extract of the *N. maculosa* eggshells (Supplementary Fig. S7) as well as in the ESI + extracted ion chromatograms corresponding to the molecular mass of **3**^**H**^ (*m/z* = 466.2 Da; Supplementary Fig. S24), we find evidence for the presence of two isomeric compounds. Indeed, the oxidative loss of any one of the terminal pyrrolic moieties in the tetrapyrrolic pigment biliverdin will produce one of the isomers, **3a**^**H**^ or **3b**^**H**^, also known as biotripyrrins a and b^33^. The occurrence of either isomer might point to their non-enzymatic origin. Furthermore, the *N. maculosa* EDTA extract also contained a small fraction of a dipyrrolic pigment with the composition C_17_H_19_N_2_O_4_ (for MH^+^). Characterized by its UV–Vis spectrum (Supplementary Fig. S7) and particularly through its well-resolved tandem MS spectrum (Supplementary Fig. S13), we assign it the formyldipyrrinone^34^ structure **5**^**H**^. It is formally derived by continued oxidative degradation of uroerythrin (Fig. 5). Whether the first biliverdin oxidation step actually takes place at the bond indicated or at the adjacent double bond, followed by the loss of the *meso*-carbon, cannot be determined, but direct and circumstantial evidence exist that at least singlet oxygen reacts specifically with the double bond^33,38^.

Bilirubin **4**^**H**^ is a well-studied catabolite of biliverdin^39^, generated through a reductive enzymatic process^40^. The adventitious formation of bilirubin from biliverdin under the oxic conditions of the pigment isolation and derivatization process is highly unlikely. This suggests that bilirubin **4**^**H**^ is a genuine eggshell pigment in *Eudromia elegans*. Since bilirubin degrades rapidly under the typical extraction conditions (converting, in part, to biliverdin, but also generating smaller (dipyrrolic), unidentified fragments, Supplementary Figs. S19–20, S22), it is understandable how bilirubin in *E. elegans* eggshells has eluded detection in the past^20, 24^. In addition, because of their structural similarity, the Raman spectra of biliverdin and bilirubin do not permit facile differentiation^41^.

Birds generally produce little bilirubin as most either lack, or have significantly reduced levels of, biliverdin reductase^42,43^. We are unaware if *E. elegans* possesses biliverdin reductase; however, work analysing the complete mitochondrial genome (GeneBank Accession no. NC_027260) of the closely related white-throated tinamou (*Tinamus guttatus*) reveals that *T. guttatus* contains genes corresponding to both biliverdin reductases A and B^44^. Moreover, the mitogenome order *of T. guttatus* is identical to that of the published mitogenome of *T. major* (GeneBank Accession no. NC_002781.3)^45^. We therefore deem it reasonable to assume that *E. elegans* can also generate bilirubin by enzymatic reduction of biliverdin.

Our colour mixing model accurately modelled the natural colours of the purple and green *N. maculosa* and *E. elegans* eggshells, respectively, by mixing the colours of the two pigments found in each eggshell. As such, this model provides supporting evidence that the extracted compounds are genuine eggshell pigments contributing toward the unique eggshell colours. Thus, tinamous have access to an expanded eggshell pigment colour palette. Furthermore, since this two-pigment model, which assumes an even mixing of the pigments throughout the pigmented layer, achieved a close match to the experimental eggshell colours, it also indicates that none of the additional mechanisms discovered to modulate eggshell colours—such as uneven distribution of the pigments throughout the cuticle and eggshell^46–48^, colour modulation by the cuticle and eggshell structure^2,3^, pigment aggregation^49^, or the strongly solvatochromic properties and conformational plasticity of linear tetrapyrroles^30,50–52^ that might express different hues in variable protein matrices—play a prominent role here. Likewise, the much less deeply yellow-coloured dipyrrins present also do not play any major role in the coloration of the eggshells.

Both novel pigments are more (photo)-labile than biliverdin. This suggests that the surface coloration for both eggs should vary substantially over time when exposed to light. Interestingly, such colour changes have been observed in tinamou eggshells and may have an adaptive function in communally nesting polyandrous birds^8^. In both species, females will lay their eggs in a male’s nest and the colour change could be useful for communally nesting females to discriminate freshly laid clutches from older clutches, thus serving as a useful cue for deciding where and when to lay^8^. Conspicuous eggshell colours may also serve as an attractive signal to other females, encouraging them to add to the clutch, which can lower predation by initiating male incubation^53^. This said, it is also plausible that these colour changes are simply non-adaptive, non-costly by-products of the underlying pigments. Further research is necessary to determine whether the (photo)-lability of these dyes has any ecological significance. The use of ephemeral, tetrapyrrole-based photo-degradable coloration to convey biological information has been proposed previously^54^.

The finding that oligopyrrolic pigments are exclusively used as eggshell pigments attests to the strong colouring ability of extended π-systems. The open-chain pigments can be derived from the degradation of haem within the shell gland of the laying bird^55^ and represent an essentially linear catabolic sequence already present elsewhere in the avian body^56^; it is a further testament that the eggshell pigments are derived by the repurposing of existing metabolic pathways^56^. The parent pigment, protoporphyrin, may itself also be derived in deviation from regular haem metabolism pathways from haem in the oviduct^57^. Future research is required to determine if the shell gland is also the synthesis site for bilirubin and uroerythrin. Interestingly, the only other study we are aware of which reports a non-porphyrin/non-biliverdin pigment (albeit without providing details or an independent verification) found another biliverdin metabolite, the tetrapyrrolic purple pigment mesobiliviolin, in the brown markings on the greenish eggshells of *Lissotis melanogaster*^19^. The finding that the eggshell pigments identified to date are oligopyrrolic pigments suggests that birds are limited to utilizing metabolites from the levulinic acid route to dye their eggs. This is in contrast to the mevalonic acid and tyrosine metabolic routes taken by the birds for the majority of the dyes to colour their plumage^58^.

## Conclusion

In conclusion, the investigation of the unusually coloured eggshells of two tinamou species revealed, next to the well-known eggshell pigment biliverdin **2**^**H**^, the presence of two hitherto unrecognized oligopyrrolic eggshell pigments: the orange tripyrrolic uroerythrin **3**^**H**^ (in eggshells of *N. maculosa*) and the brown-yellow tetrapyrrolic bilirubin **4**^**H**^ (in eggshells of *E. elegans*), both isolated and identified as their diacids and their dimethyl esters. A colour mixing model supports the conclusion that the eggshell colours of both species are generated by the presence of the previously identified pigment biliverdin in combination with the two previously unknown colourants*.* Notably, the chocolate-brown coloration of the *E. elegans* eggshells can be achieved without any contribution of the traditional brown pigment, protoporphyrin IX. The yellow–brown bilirubin and orange uroerythrin thus expand the pallete of the known eggshell pigments. Furthermore, we suggest that the layering of biliverdin with these pigments possessing different abilities to photo-degrade may have an adaptive value for the tinamou species investigated.

## Methods

### Materials

The *N. maculosa* eggshell from captive-bred birds (Chile) were sourced from The Eggery Place (https://theeggeryplace.com). The *E. elegans* eggshells came from the Peabody Museum collection. All solvents used were HPLC or spectroscopy grade and used as provided by commercial suppliers. Protoporphyrin IX **1**^**H**^, its dimethyl ester **1**^**Me**^ , biliverdin IXα **2**^**H**^, its dimethyl ester **2**^**Me**^, bilirubin **4**^**H**^, its dimethyl ester **4**^**Me**^^28^ were either provided by Porphyrin Products, Logan, UT, or were extracted from hen (**1**^**Me**^)^27^ or emu eggshells (**2**^**Me**^)^28^, or chemical reduction of biliverdin **2**^**Me**^, respectively (**4**^**Me**^)^28^.

### Acid-based eggshell extraction

A slightly modified version of the classic eggshell pigments extraction protocol using methanolic H_2_SO_4_ solution was used^25–28^. A detailed protocol is provided in the ESI.

### EDTA-based eggshell extraction

The EDTA pigment extractions were performed as described in Gorchein et al.^21^. A detailed protocol is provided in the ESI.

### Instrumentation

#### UV–Vis spectroscopy

We either used a Cary 50 spectrometer or the Agilent 1,100 series HPLC UV-detector to record the UV–Vis spectra of the fractions and mixtures in the solvents indicated.

#### NP-HPLC

A portion of the *N. maculosa* eggshell extracts and the blue-green, non-polar band of the *E. elegans* eggshells were dissolved in ethyl acetate (~ 1 mL) and analysed using an Agilent 1,100 series HPLC (equipped with a Grace analytical normal-phase Apollo silica column, 4.6 × 250 mm, 5 μm and autosampler). The mobile phase employed a gradient delivery of hexanes and EtOAc: linear gradient of pure hexanes to 70:30 v/v hexanes:EtOAc over 6 min, then isocratic delivery of 70:30 v/v hexanes:EtOAc over 7 min, followed by linear gradient to pure ethyl acetate over 2 min, all with a flow rate of 1.5 mL/min. The detection wavelengths of the UV–Vis detector were set to 400 nm and 475 nm. The polar, green band of the *E. elegans* eggshell extracts were dissolved in MeOH (~ 1 mL) and analysed using the setup described above but using an isocratic delivery of 100% MeOH. The detection wavelength of the UV–Vis detector was set to 400 nm.

#### HR-MS

High-resolution mass spectra were recorded on a Thermo Scientific Q Exactive Quadrupole-Orbitrap mass spectrometer in the ESI + mode using 100% acetonitrile.

#### RP–HPLC–MS/MS

The dried EDTA eggshells extracts from the EDTA extraction were dissolved in MeOH (100 µL), centrifuged at 13,000 rpm, and transferred to LC–MS vials. RP-HPLC-ESI^+^-MS was performed using a SCIEX ExionLC (using a Kinetex C18 Reversed-Phase Column, 100 × 2.1 mm) coupled to a SciEX X500B QTof mass spectrometer. The samples were separated employing a 30 min gradient delivery of 5:95 to 98:2 ACN (+ 0.01% TFA) to water (+ 0.01% TFA). The measurements were contrasted against those using biliverdin **2**^**H**^ and bilirubin **4**^**H**^ standards.

### Colour mixing model

Details to the colour mixing model used are provided in the ESI.

## Supplementary information

Supplementary information

## Data Availability

All data is available upon request.
