# Supplementary material for: Expanding the eggshell colour gamut: uroerythrin and bilirubin from tinamou (Tinamidae) eggshells
Source: Sci Rep. 2020 Jul 9;10:11264. doi: 10.1038/s41598-020-68070-7 (PMC7347609; doi:10.1038/s41598-020-68070-7)
Supplement: Supplementary file 1 — Supplementary information [file 41598_2020_68070_MOESM1_ESM.pdf]

Supporting Information (SI) to:

Expanding the eggshell colour gamut: Uroerythrin and bilirubin from tinamou (*Tinamidae*) eggshells

Randy Hamchand[1], Daniel Hanley[2], Richard O. Prum[3] & Christian Brückner[1]

1. Department of Chemistry,
University of Connecticut, Unit 3060, Storrs, CT 06269-3060, USA

2. Department of Biology and Environmental Sciences,
Long Island University – Post, Brookville, NY, 11548, USA

3. Department of Ecology and Evolutionary Biology, and Peabody Museum of Natural History,
Yale University, New Haven, CT 06520-8105, USA

Correspondence and requests for materials should be addressed to C.B.
(email: c.bruckner@uconn.edu, FAX +1-860-486-2981)

SI Table of Contents

H_2SO_4/MeOH-based Pigment Extraction Protocol with Concomitant Esterification: S4

Pigment Concentration Protocol: S4

EDTA-based Eggshell Extraction Protocol S5

Purple *Nothura maculosa* Eggshell Extractions – Acidic Conditions S6

Figure S1. Benchmark data: Left: HPLC chromatogram of biliverdin dimethyl ester **2**^{Me} extracted from emu eggshells (400 nm detection wavelength). Right: Normalized UV-vis spectrum of biliverdin dimethyl ester **2**^{Me} (in ethyl acetate) extracted from emu eggshells. S6

Figure S2. Top: HPLC chromatogram of *N. maculosa* extract (475 nm detection wavelength). Bottom: UV-vis spectra (ethyl acetate) of the *N. maculosa* extract fractions occurring at the times indicated. S6

Figure S3. HR ESI+-MS spectrum (100% CH_3CN) of commercial biliverdin dimethyl ester **2**^{Me}. S7

Figure S4. HR ESI+-MS spectrum (100% CH_3CN) of 18.0 min peak (**2**^{Me}) from the H_2SO_4/MeOH extraction of *N. maculosa* eggshells.. .... S7

Figure S5. HR ESI+-MS (TOF, 100% MeOH) of 21.1 min peak (assigned to uroerythrin dimethyl ester **3**^{Me}) extracted using H_2SO_4/MeOH from *N. maculosa* eggshells. S8

Figure S6. HR ESI+-MS^2 spectrum (TOF, 100% MeOH) of the *m/z* 516.2 Da peak of the 21.1 min peak (assigned to uroerythrin dimethyl ester **3**^{Me}) extracted using H_2SO_4/MeOH from *N. maculosa* eggshells S8

Purple *Nothura maculosa* Eggshell Extractions – EDTA Conditions S9

Figure S7. RP-HPLC Chromatogram at 475 nm (red trace) and 400 nm (blue trace) and (bottom) the UV-vis spectra of the fractions indicted in the chromatograms S9

Figure S8. RP-HPLC HR ESI+-MS^2 spectrum (QTOF, 100% MeOH) of the *m/z* peak at 583.2 Da of a commercial biliverdin diacid sample of **2**^{H} S10

Figure S9. RP-HPLC HR ESI+-MS^2 spectrum (QTOF, 100% MeOH) of the 583.2 Da m/z peak of **2**^{H} extracted from *N. maculosa* eggshells using the EDTA extraction protocol. S10

Figure S10. RP-HPLC HR ESI+-MS^2 spectrum (TOF, 100% MeOH) of the 585.3 Da m/z peak of a commercial sample of bilirubin **4**^{H}. S11

Figure S11. RP-HPLC HR ESI+-MS^2 spectrum (QTOF, 100% MeOH) of the 585.3 Da peak of **4**^{H} extracted from *N. maculosa* eggshells using the EDTA extraction protocol S11

Figure S12. RP-HPLC HR ESI+-MS^2 spectrum (QTOF, 100% MeOH) of the 466.2 Da peak of **3**^{H} extracted from *N. maculosa* eggshells using the EDTA extraction protocol, and possible fragmentation scheme based on typical fragmentations observed in linear naturally occurring oligopyrroles. S12

Figure S13. RP-HPLC HR ESI+-MS^2 spectrum (QTOF, 100% MeOH) of 315.1 Da peak extracted from *N. maculosa* eggshells using the EDTA extraction protocol, and possible fragmentation scheme based on typical fragmentations observed in linear naturally occurring oligopyrroles. S13

Green *Eudromia elegans* Eggshell Extractions – Acidic Conditions S14

Figure S14. HPLC trace of raw *E. elegans* eggshell extract S14

Figure S15. Left: HPLC comparison of commercial bilirubin dimethyl ester **4^Me^** and the polar fraction of the H_2SO_4/MeOH extraction of *E. elegans* eggshells (left). Right: UV-vis spectra (ethyl acetate) of commercial bilirubin dimethyl ester **4^Me^** and the *E. elegans* eggshell extraction polar fraction. S14

Figure S16. HR ESI+-MS spectrum (100% CH_3OH) of **2^Me^** from the low polarity fraction of the H_2SO_4/MeOH extraction of *E. elegans* eggshells. S15

Figure S17. HR ESI+-MS spectra (MeOH) of commercial **4^Me^**. S16

Figure S18. HR ESI+-MS spectrum (CH_3CN) of **4^Me^** extracted with H_2SO_4/MeOH from *E. elegans* eggshells. S17

Green *Eudromia elegans* Eggshell Extractions – EDTA Conditions S18

Figure S19. RP-HPLC Chromatogram at 400 nm and (bottom) the UV-vis spectra of the fractions indicted in the chromatogram. S18

Figure S20. RP-HPLC HR ESI+-MS^2 spectrum (QTOF, 100% MeOH) (100% MeOH) of peak A extracted from *E. elegans* eggshells using the EDTA extraction protocol S19

Figure S21. RP-HPLC HR ESI+-MS^2 spectrum (QTOF, 100% MeOH) of **2^H^** extracted from *E. elegans* eggshells using the EDTA extraction protocol S19

Figure S22. RP-HPLC HR ESI+-MS^2 spectrum (QTOF, 100% MeOH) of peak C extracted from *E. elegans* eggshells using the EDTA extraction protocol. S20

Figure S23. RP-HPLC HR ESI+-MS^2 spectrum (QTOF, 100% MeOH) of bilirubin **4^H^** extracted from *E. elegans* eggshells using the EDTA extraction protocol S20

Figure S24. ESI+ extracted ion chromatograms (EICs) of LC-MS traces for crude EDTA eggshell extracts, biliverdin diacid standard, and bilirubin diacid standard. S21

Colour Mixing Modelling S22

References for Supporting Information S24

H_2SO_4/MeOH-based Pigment Extraction Protocol with Concomitant Esterification:

A slightly modified version of the classic eggshell pigments extraction protocol using methanolic H_2SO_4 solution was used:[1-3] Eggshells (100-1000 mg) were weighted and ground into a fine powder in a porcelain mortar. The powder was placed in a 25 ml round-bottom flask equipped with a magnetic stir bar and reflux condenser. To this was added a methanolic H_2SO_4 solution (1.8 M, 5%; 5 mL). The mixture was heated on a heating mantle to a gentle reflux for 45 min. The pH of the cooled solution was adjusted to between 6-8 by addition of sat. aq. $NaHCO_3$ (~3.5 mL) until the effervescence stopped. The suspension was then filtered through a small (8 cm diameter) Büchner funnel and the filter cake was washed with ethyl acetate (EtOAc) (~10 mL). The filtrate was transferred to a 50 mL separatory funnel containing water (10 mL). The aqueous layer was extracted with EtOAc (2 × 5 mL). The combined organic layers were dried over anhydrous Na_2CO_3 (~ 1 g), the drying agent was removed by gravity filtration, and the clear but coloured solution was concentrated by rotary evaporation. The volume of the organic fraction was determined at this point for the determination of the optical pigment concentration. The *E. elegans* extract was passed through a short silica gel column (0.5 × 3 cm). The nonpolar main blue-green band was collected (EtOAc) and the eluent changed (to 4:1 EtOAc:MeOH) to elute a yellow-brown fraction. The fractions were evaporated to dryness under a stream of N_2 and stored at –20˚C.

We verified that the dye extraction and esterification protocol did not trigger the degradation of protoporphyrin $\mathbf{1}^{\mathbf{H}}$ or biliverdin $\mathbf{2}^{\mathbf{H}}$, thereby generating artefacts by applying independently sourced protoporphyrin $\mathbf{1}^{\mathbf{H}}$ or biliverdin $\mathbf{2}^{\mathbf{H}}$, dissolved in MeOH, onto commercial (non-waxed) white hen eggshells. We then submitted the dyed eggshells to the same extraction protocol as the tinamou eggshells described above and verified the presence of the pigments, as their dimethyl esters, $\mathbf{1}^{\mathbf{Me}}$ or $\mathbf{2}^{\mathbf{Me}}$, and the absence of any notable degradation products by HPLC.

Pigment Concentration Protocol:

The estimation of the quantity and molar ratios of the pigments was based on the known extinction coefficients for biliverdin dimethyl ester $\mathbf{2}^{\mathbf{Me}}$ (in EtOH, λ = 377 nm, ε = 56,200 $cm^{-1}M^{-1}$)[4], uroerythrin dimethyl ester monomethyl ether $\mathbf{3}^{\mathbf{MeOMe}}$ (in MeOH, λ = 271 nm, λ = 330 nm, ε = 32,000 $cm^{-1}M^{-1}$; λ = 523 nm ε = 27,000 $cm^{-1}M^{-1}$)[5], bilirubin dimethyl ester $\mathbf{4}^{\mathbf{Me}}$ (in $CHCl_3$, λ = 451 nm, ε = 54,000 $cm^{-1}M^{-1}$)[4,6], and those of a tripyrrinedione closely related to $\mathbf{3}^{\mathbf{H}}$[7]. The extinction coefficients for $\mathbf{3}^{\mathbf{H}}$ or $\mathbf{3}^{\mathbf{Me}}$ have not been reported but can reasonably be assumed to be essentially identical since the esterification does not affect the chromophore; furthermore,

we assumed the extinction coefficients for $\mathbf{3}^{\mathbf{H}}$ and $\mathbf{3}^{\mathbf{MeOMe}}$ at 271 nm (the wavelength least variant upon chromophore etherification) to be identical. Solvatochromic effects were ignored.

EDTA-based Eggshell Extraction Protocol

The EDTA pigment extractions were performed as described in Gorchein *et al.*:[8] Briefly, eggshell (170 mg) were crushed into small fragments and placed in 1.5 mL Eppendorf centrifuge tubes. An aqueous disodium EDTA solution (1.5 mL, 100 mg/mL, pH 7.2) was added and vortexed for 1 min. The tubes were then uncapped and incubate in the EDTA solution for additional 5 min. Once the effervescence ceased, the samples were centrifuged at 14,000 g for 1 min and the supernatants were discarded. This procedure was repeated three times. An acetonitrile (ACN)-glacial acetic acid (AcOH) solution (4:1, v/v) was added to the final pellet and the mixture was repeatedly vortexed and uncapped in 30 s intervals for 2 min. The supernatants were transferred to new tubes, protected from light with tin foil and dried to a film under a stream of nitrogen in the dark.

Purple *Nothura maculosa* Eggshell Extractions – Acidic Conditions

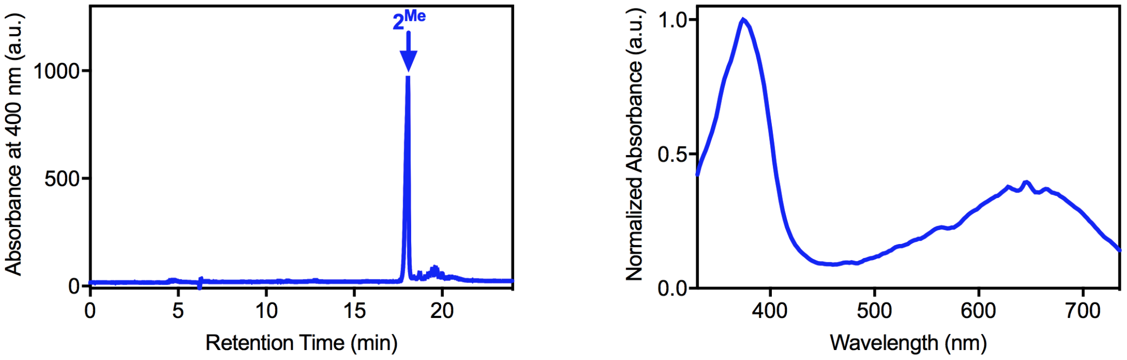

Figure S1. Benchmark data: Left: HPLC chromatogram of biliverdin dimethyl ester **2**$^{\mathbf{Me}}$ extracted from emu eggshells (400 nm detection wavelength). The splitting of the peak for biliverdin is an indication of the presence of geometric isomers.[9] Right: Normalized UV-vis spectrum of biliverdin dimethyl ester **2**$^{\mathbf{Me}}$ (in ethyl acetate) extracted from emu eggshells.

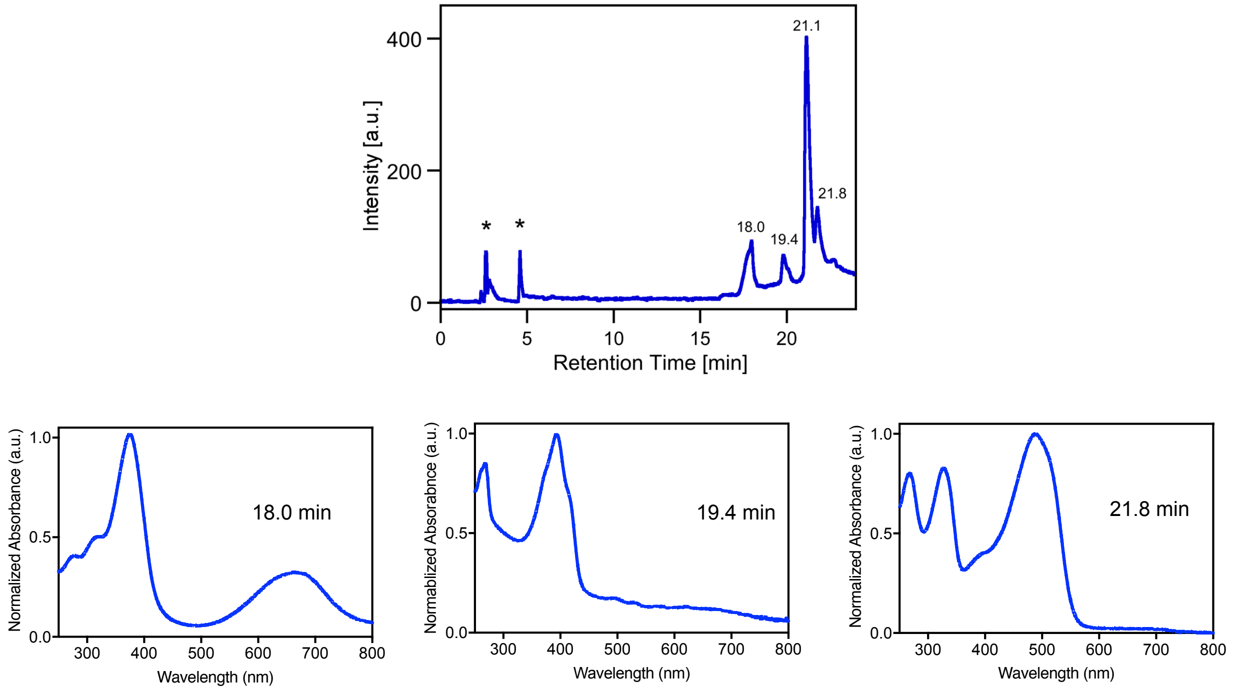

Figure S2. Top: HPLC chromatogram of *N. maculosa* extract (475 nm detection wavelength; for protocol see Methods Section; * indicate artefacts). Bottom: UV-vis spectra (ethyl acetate) of the *N. maculosa* extract fractions occurring at the times indicated. 18.0 min: biliverdin dimethyl ester **2**$^{\mathbf{Me}}$. 19.4 min: dipyrrinone; a suitable MS of this compound could not be obtained but cf. to its acid form (Figure S7A and Figure S13). 21.1 + 21.8 min: uroerythrin dimethyl ester **3**$^{\mathbf{Me}}$, two isomers; for the presence of two isomers of uroerythrin in the eggshells, see Figure S24.

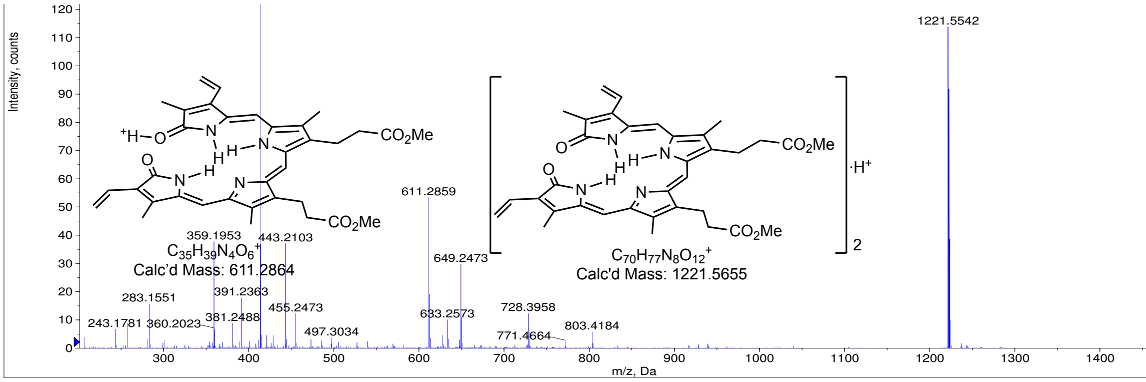

Figure S3. HR ESI+-MS spectrum (100% CH_3CN) of commercial biliverdin dimethyl ester **2^{Me}**.

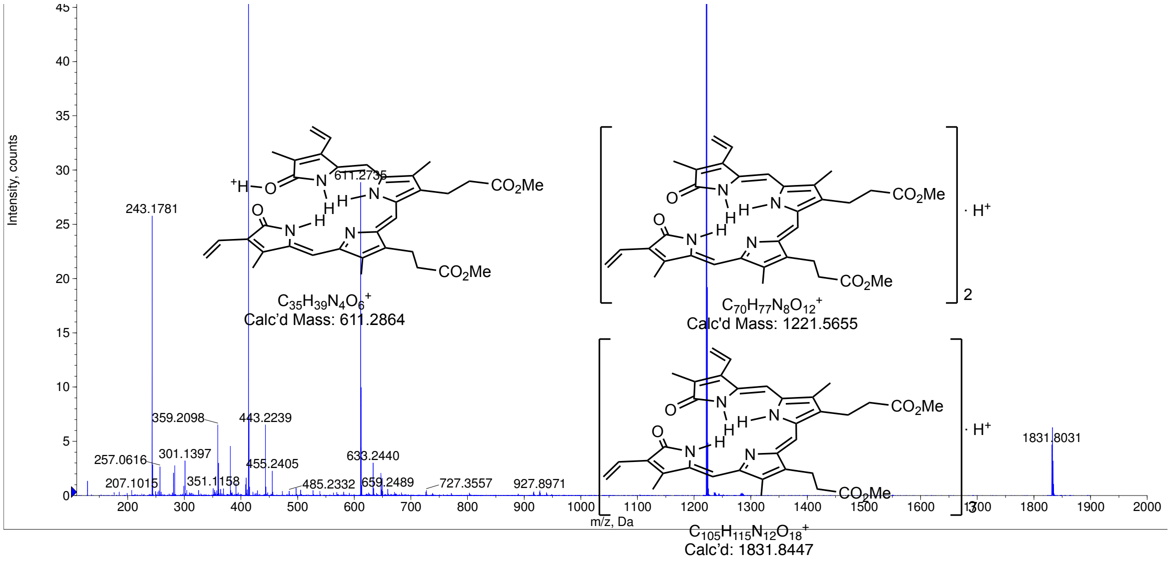

Figure S4. HR ESI+-MS spectrum (100% CH_3CN) of 18.0 min peak (**2^{Me}**) from the H_2SO_4/MeOH extraction of *N. maculosa* eggshells. For a comparison to a commercial sample, cf. Figure S3.

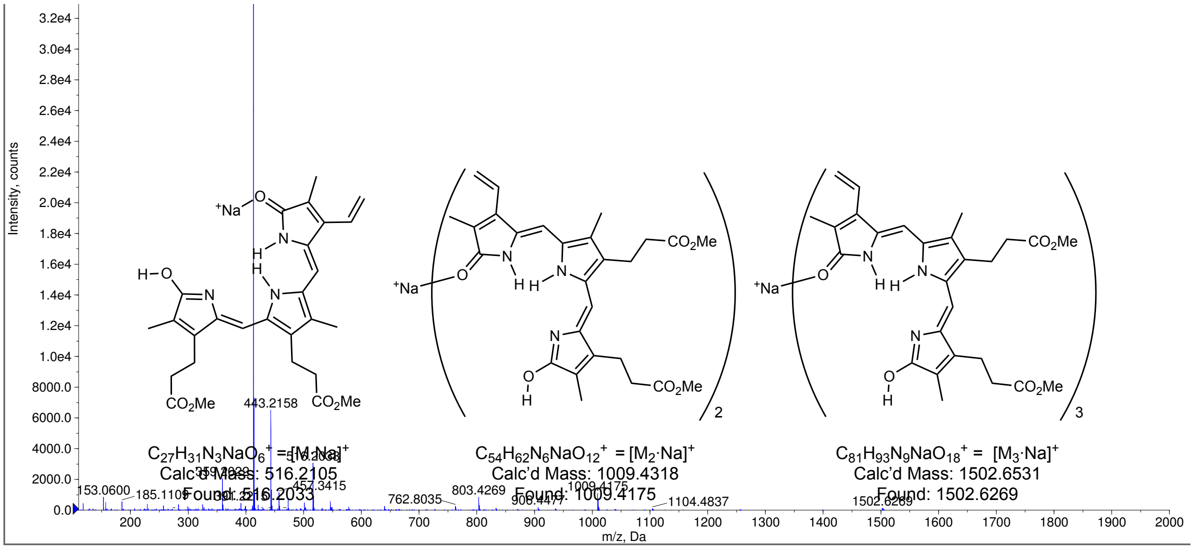

Figure S5. HR ESI+-MS (TOF, 100% MeOH) of 21.1 min peak (assigned to uroerythrin dimethyl ester **3^{Me}**) extracted using H_2SO_4/MeOH from *N. maculosa* eggshells.

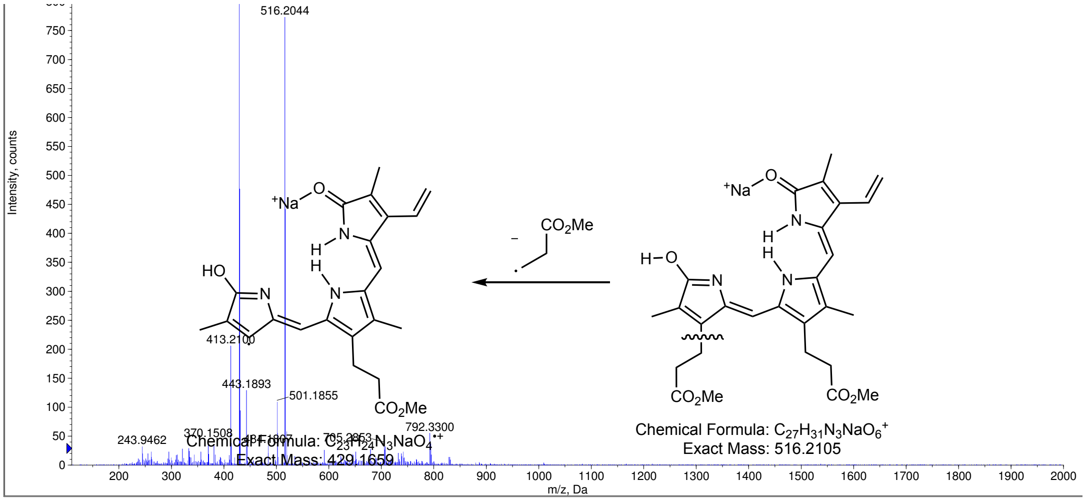

Figure S6. HR ESI+-MS^2 spectrum (TOF, 100% MeOH) of the *m/z* 516.2 Da peak of the 21.1 min peak (assigned to uroerythrin dimethyl ester **3^{Me}**) extracted using H_2SO_4/MeOH from *N. maculosa* eggshells; collision energy of 40 V.

Purple *Nothura maculosa* Eggshell Extractions – EDTA Conditions

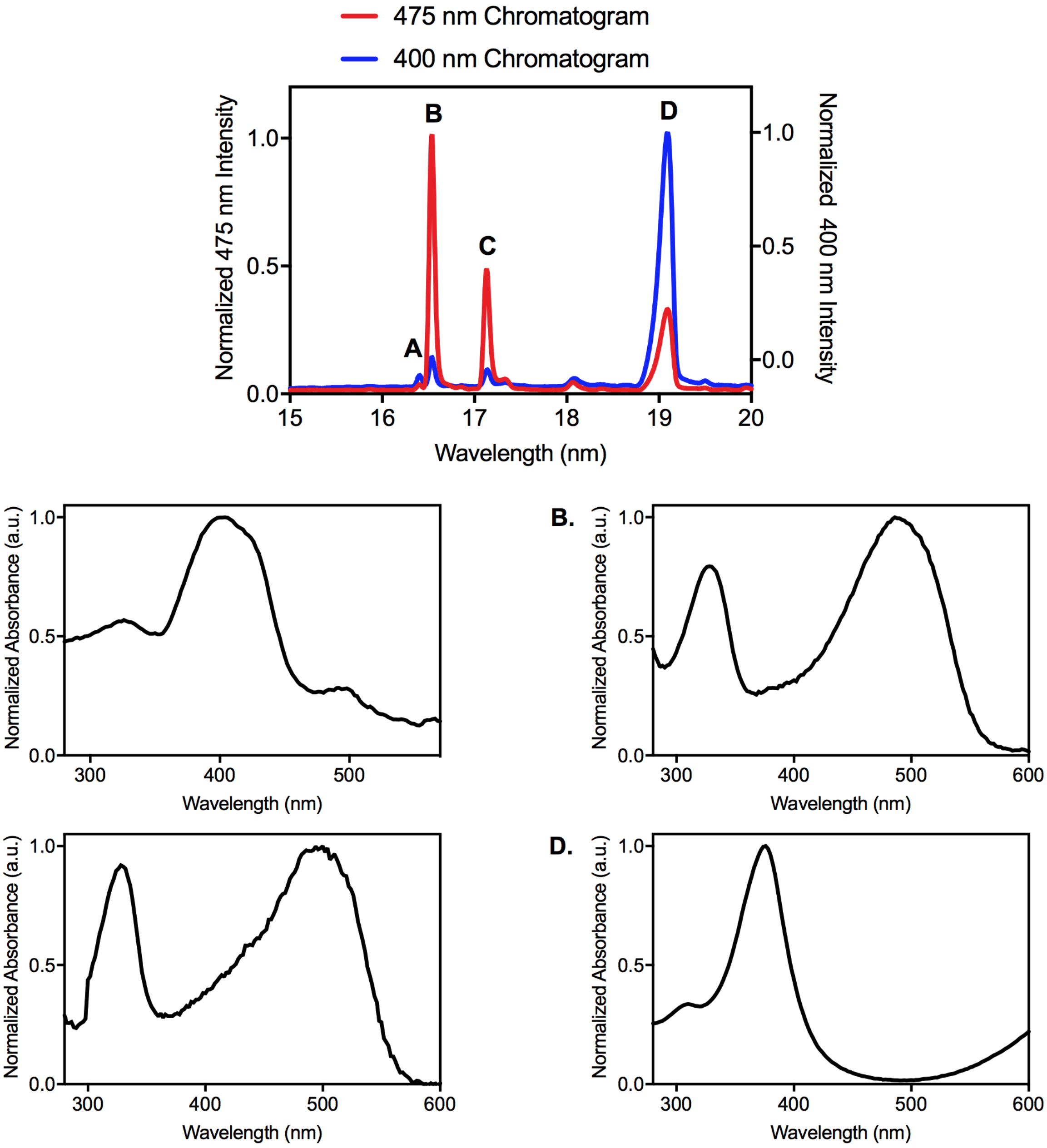

Figure S7. RP-HPLC Chromatogram at 475 nm (red trace) and 400 nm (blue trace) and (bottom) the UV-vis spectra of the fractions indicted in the chromatograms: A: presumed dipyrrinone with low resolution mass of 315.1 m/z (cf. to Figure S2) B: uroerythrin – isomer 1 C: uroerythrin – isomer 2; for the presence of two uroerythrin isomers[9] (cf. to Figure S24). D: biliverdin dimethyl ester $\mathbf{2}^{Me}$.

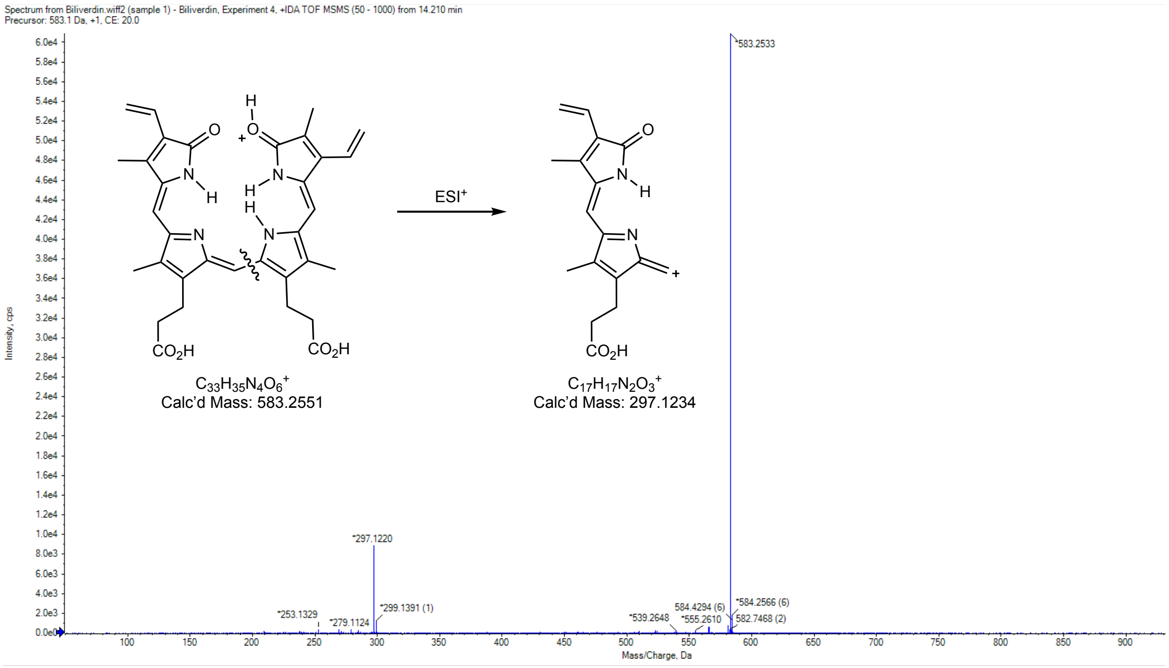

Figure S8. RP-HPLC HR ESI+-MS^2 spectrum (QTOF, 100% MeOH) of the 583.2 Da *m/z* peak of commercial biliverdin diacid sample $\mathbf{2^H}$; collision energy 20 V.

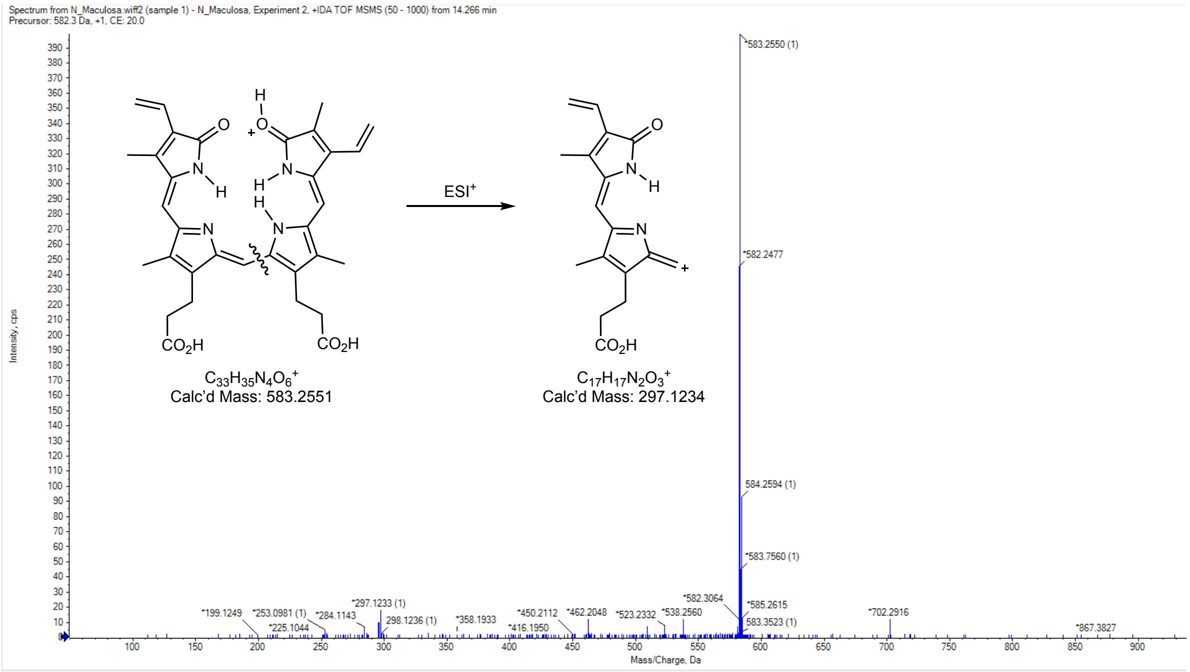

Figure S9. RP-HPLC HR ESI+-MS^2 spectrum (QTOF, 100% MeOH) of the 583.2 Da *m/z* peak of $\mathbf{2^H}$ extracted from *N. maculosa* eggshells using the EDTA protocol; collision energy 20 V.

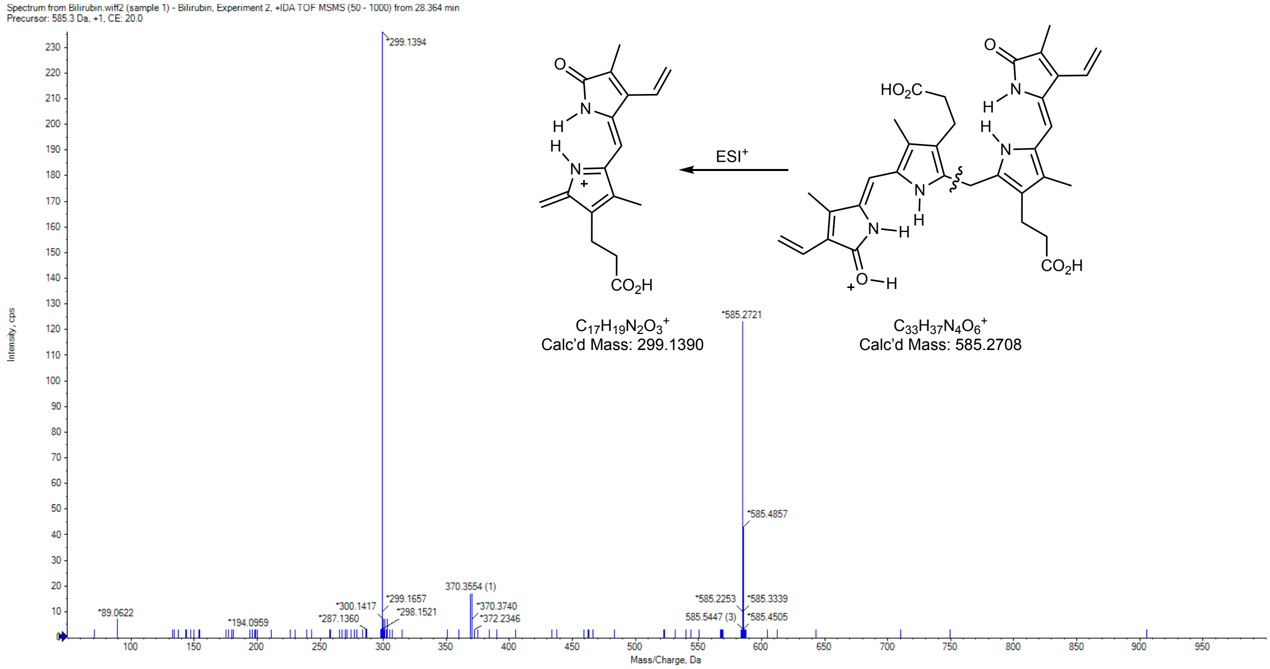

Figure S10. RP-HPLC HR ESI+-MS^2 spectrum (QTOF, 100% MeOH) of the 585.3 Da m/z peak of a commercial sample of bilirubin **4^H**; collision energy 20 V.

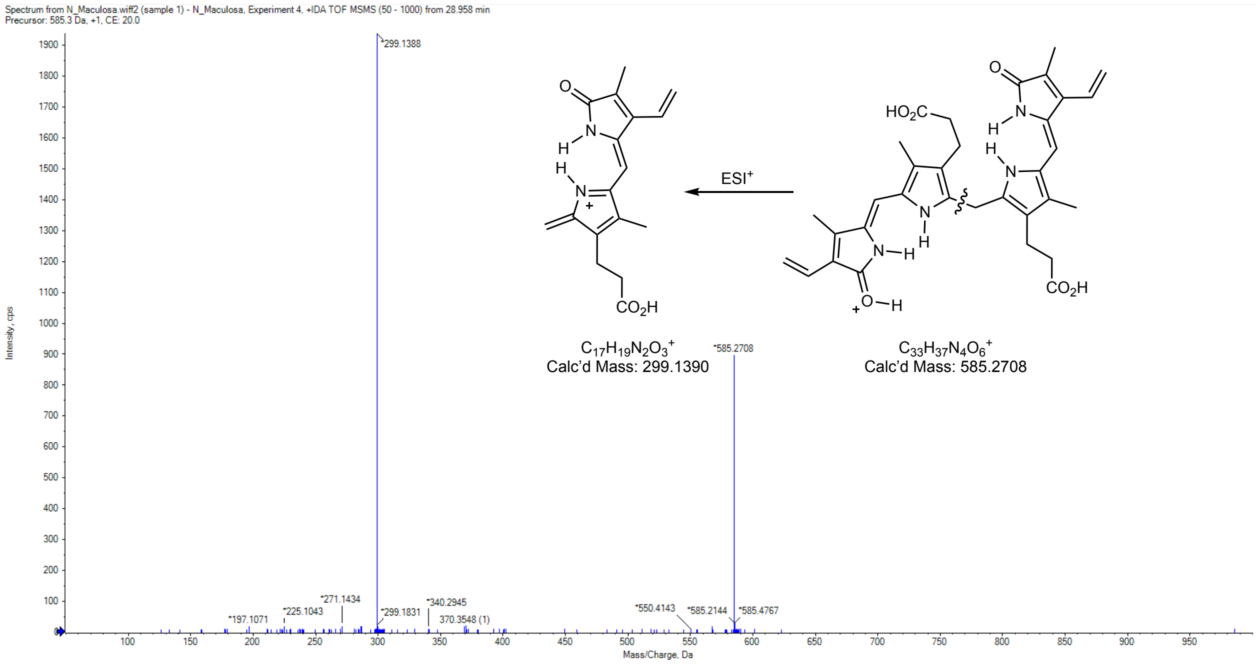

Figure S11. RP-HPLC HR ESI+-MS^2 spectrum (QTOF, 100% MeOH) of the 585.3 Da peak of **4^H** extracted from *N. maculosa* eggshells using the EDTA extraction protocol; collision energy 20 V.

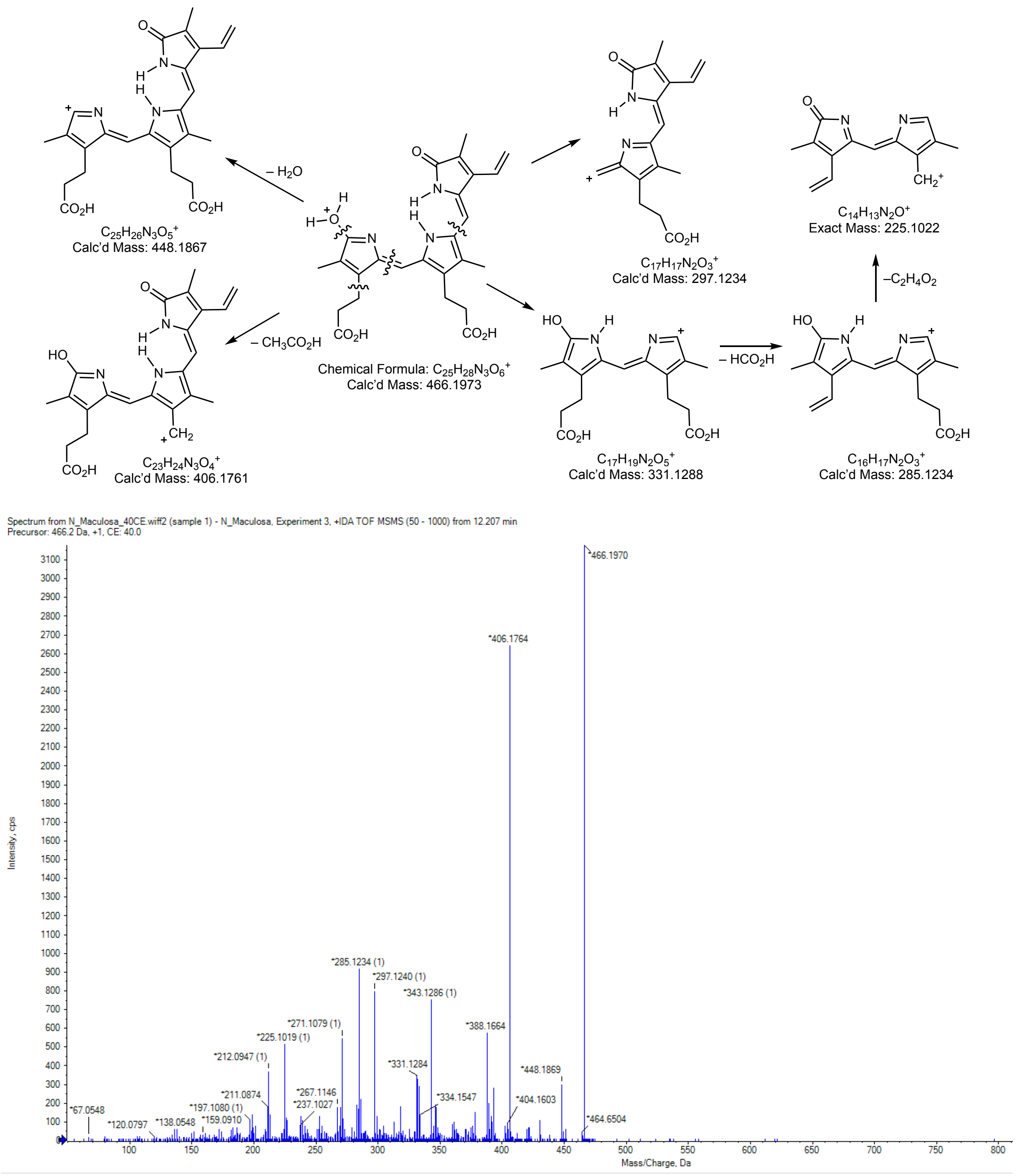

Figure S12. RP-HPLC HR ESI+-MS^2 spectrum (QTOF, 100% MeOH) of the 466.2 Da peak of $\mathbf{3^H}$ extracted from *N. maculosa* eggshells using the EDTA extraction protocol (cf. Figure S7B), and possible fragmentation scheme based on typical fragmentations observed in linear naturally occurring oligopyrroles.[10]

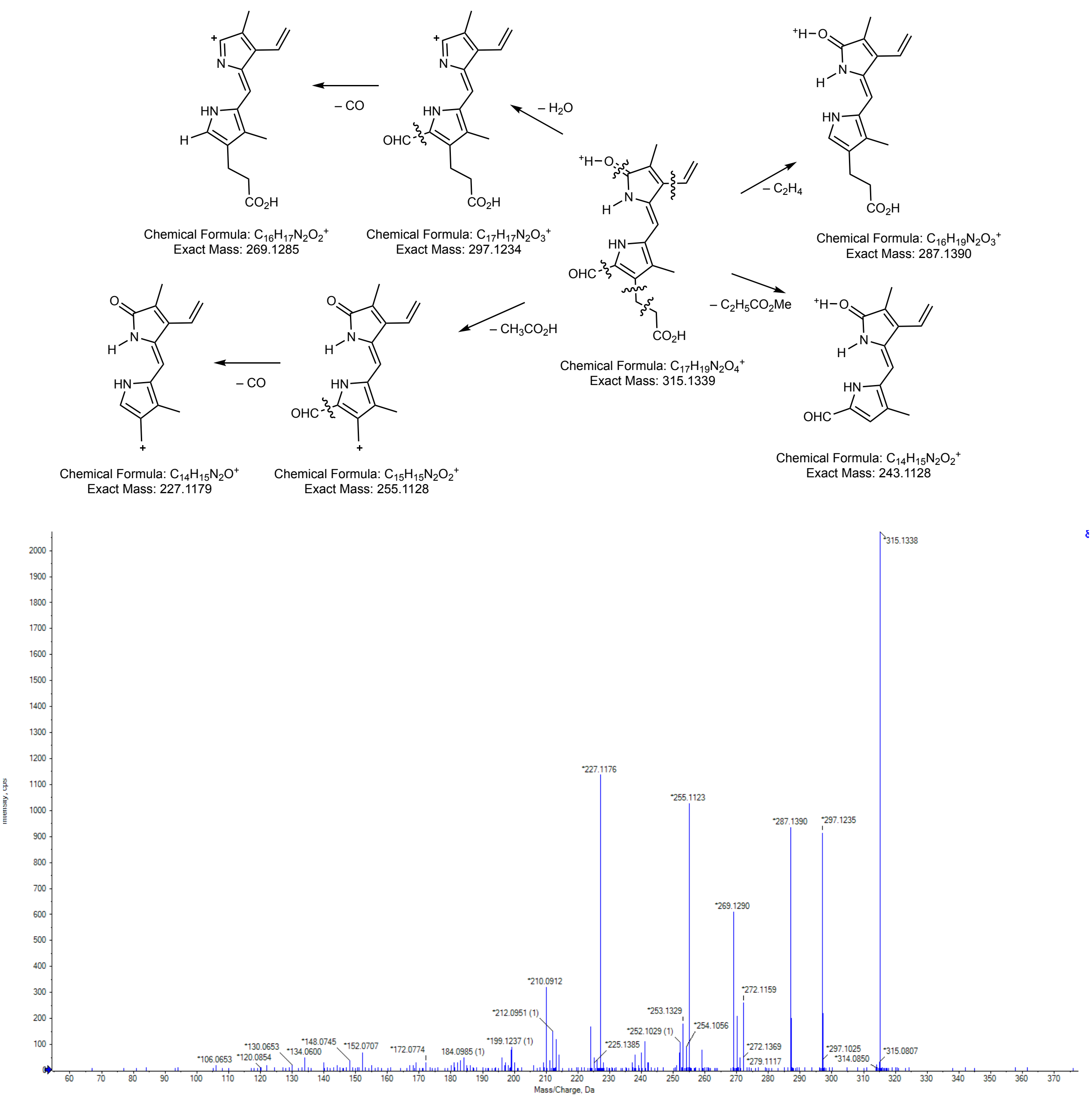

Figure S13. RP-HPLC HR ESI+-MS^2 spectrum (QTOF, 100% MeOH) of 315.1 Da peak extracted from *N. maculosa* eggshells using the EDTA extraction protocol (cf. Figure S7A), and possible fragmentation scheme based on typical fragmentations observed in linear naturally occurring oligopyrroles. The fragmentation of the compound matches that of the dipyrrole oxidation products of biliverdin.[10]

Green *Eudromia elegans* Eggshell Extractions – Acidic Conditions

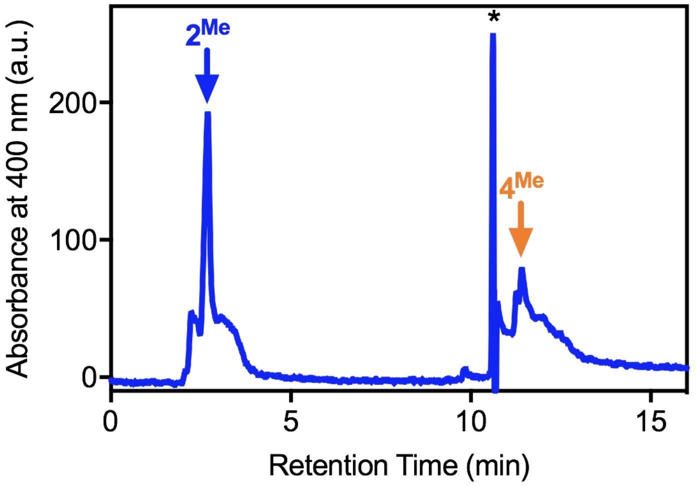

Figure S14. HPLC trace of raw *E. elegans* eggshell. HPLC employed an isocratic delivery of ethyl acetate over 5 min, followed by a linear gradient from ethyl acetate to 50:50 v/v ethyl acetate: methanol over 5 min; 50:50 ethyl acetate: methanol was maintained until the completion of the run. * indicates artefact.

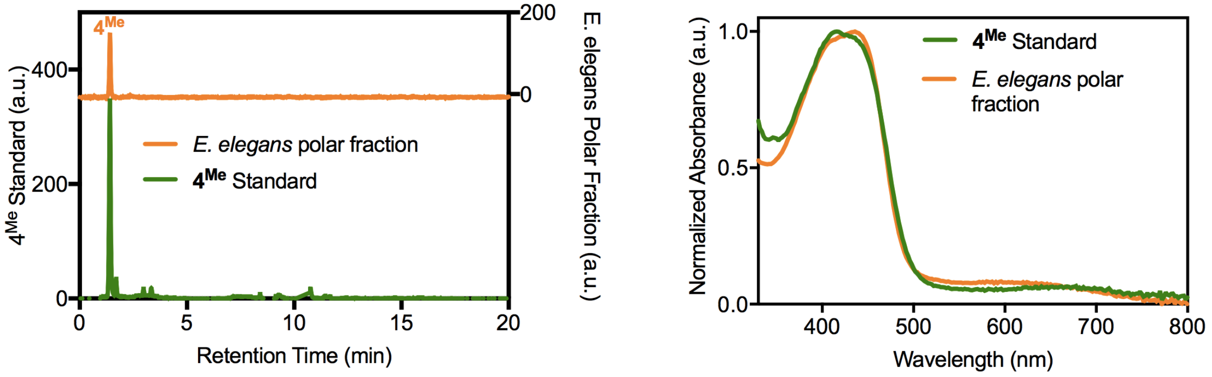

Figure S15. Left: HPLC comparison of commercial bilirubin dimethyl ester **4**^{Me} and the polar fraction of the H_2SO_4/MeOH extraction of *E. elegans* eggshells (left). Right: UV-vis spectra (ethyl acetate) of commercial bilirubin dimethyl ester **4**^{Me} and the *E. elegans* eggshell extraction polar fraction.

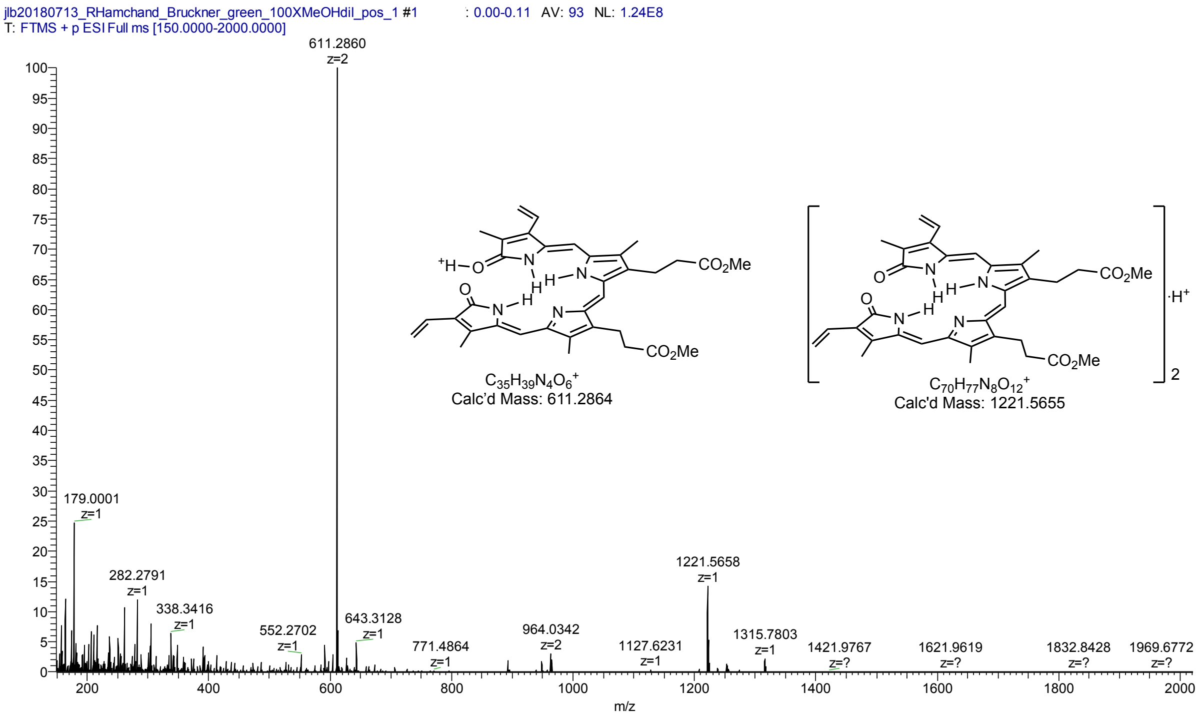

Figure S16. HR ESI+-MS spectrum (100% CH_3OH) of $\mathbf{2}^{\mathbf{Me}}$ from the low polarity fraction of the H_2SO_4/MeOH extraction of *E. elegans* eggshells. Cf. to Figure S3 for MS of commercial biliverdin dimethyl ester $\mathbf{2}^{\mathbf{Me}}$.

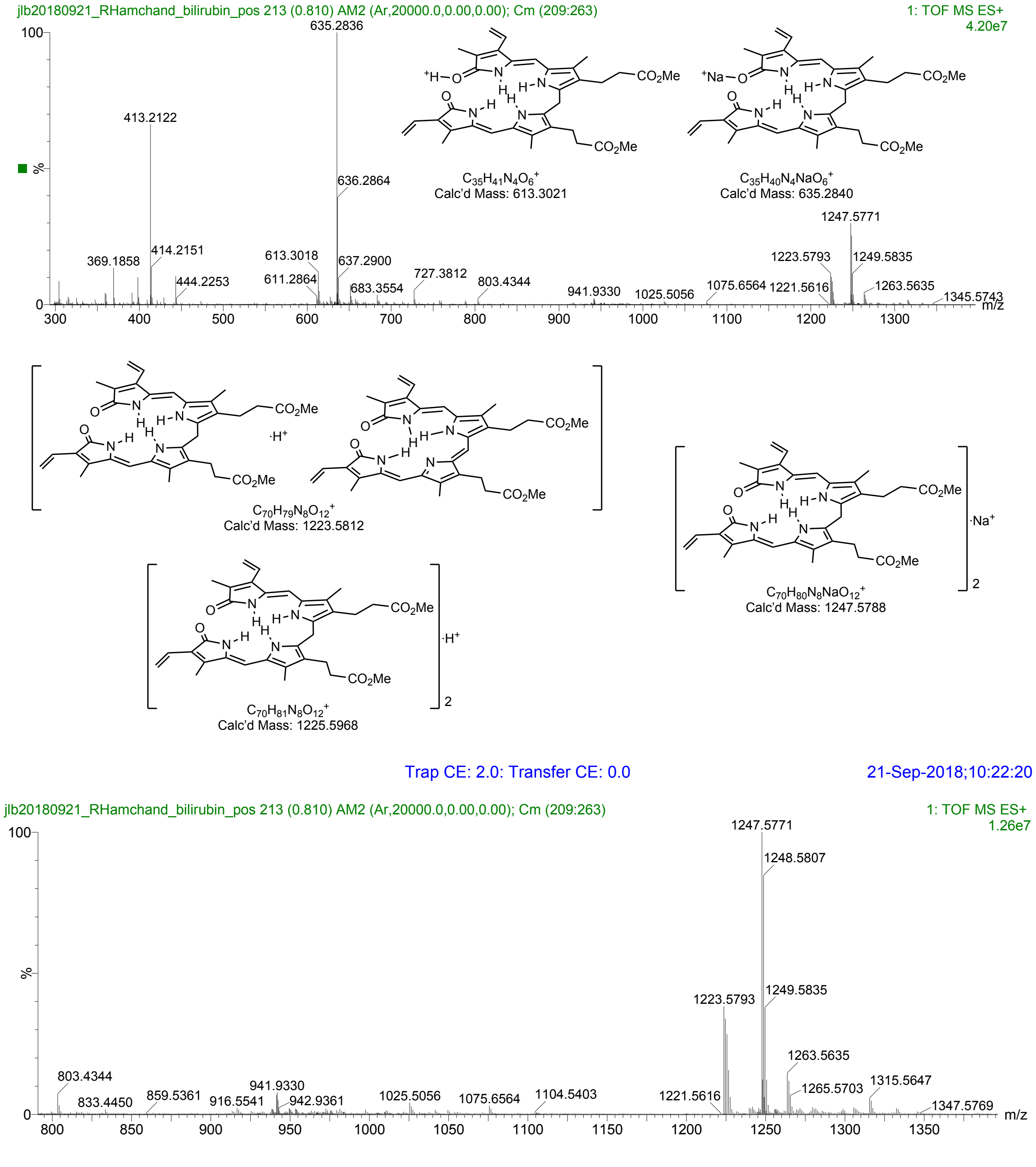

Figure S17. HR ESI+-MS spectra (MeOH) of commercial **4**^{Me}. Note: Traces of biliverdin dimethyl ester **3**^{Me} are also present; *inter alia* visible at *m/z* = 611.2864 (cf. to Figure S3) and the formation of the mixed dimer at *m/z* = 1223.5793.

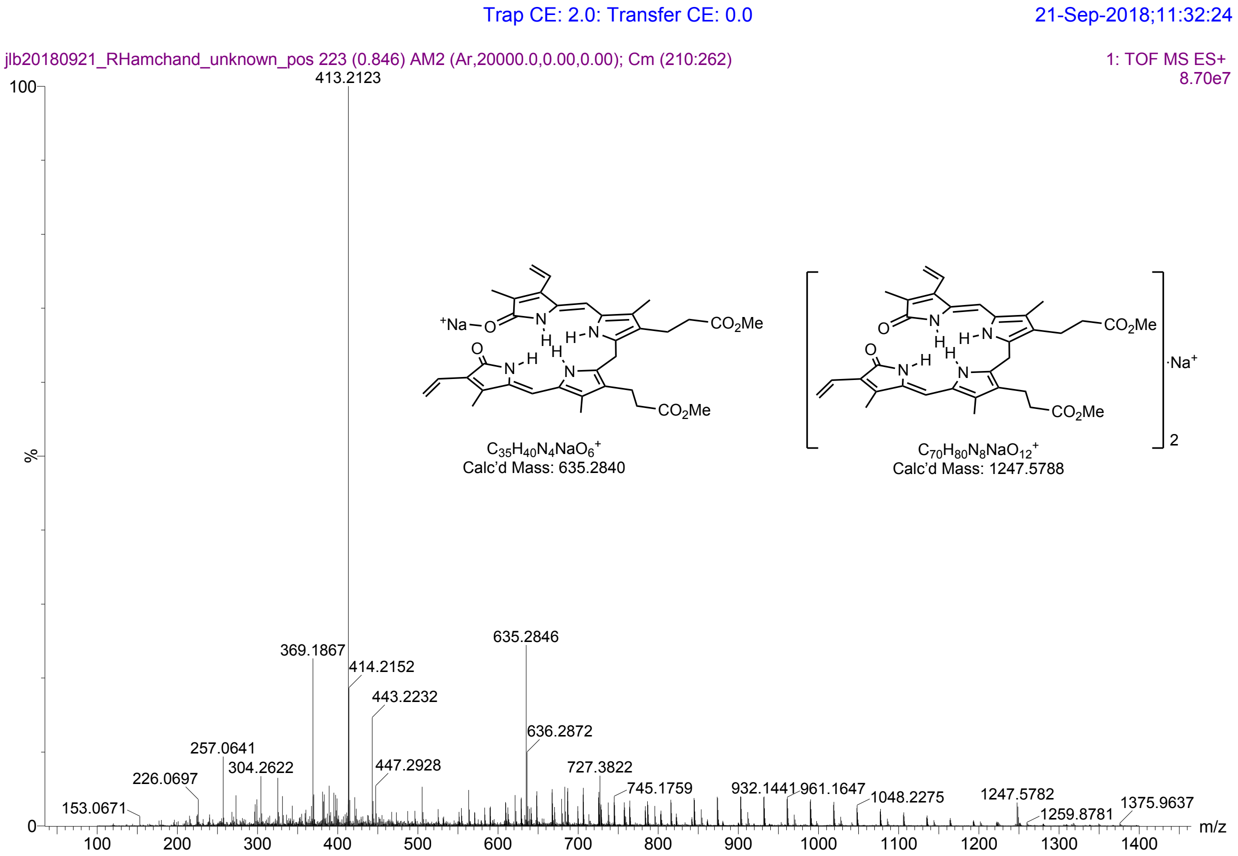

Figure S18. HR ESI+-MS spectrum (CH_3CN) of **4^{Me}** extracted with H_2SO_4/MeOH from *E. elegans* eggshells. For a comparison to a commercial sample, cf Fig. S17.

Green *Eudromia elegans* Eggshell Extractions – EDTA Conditions

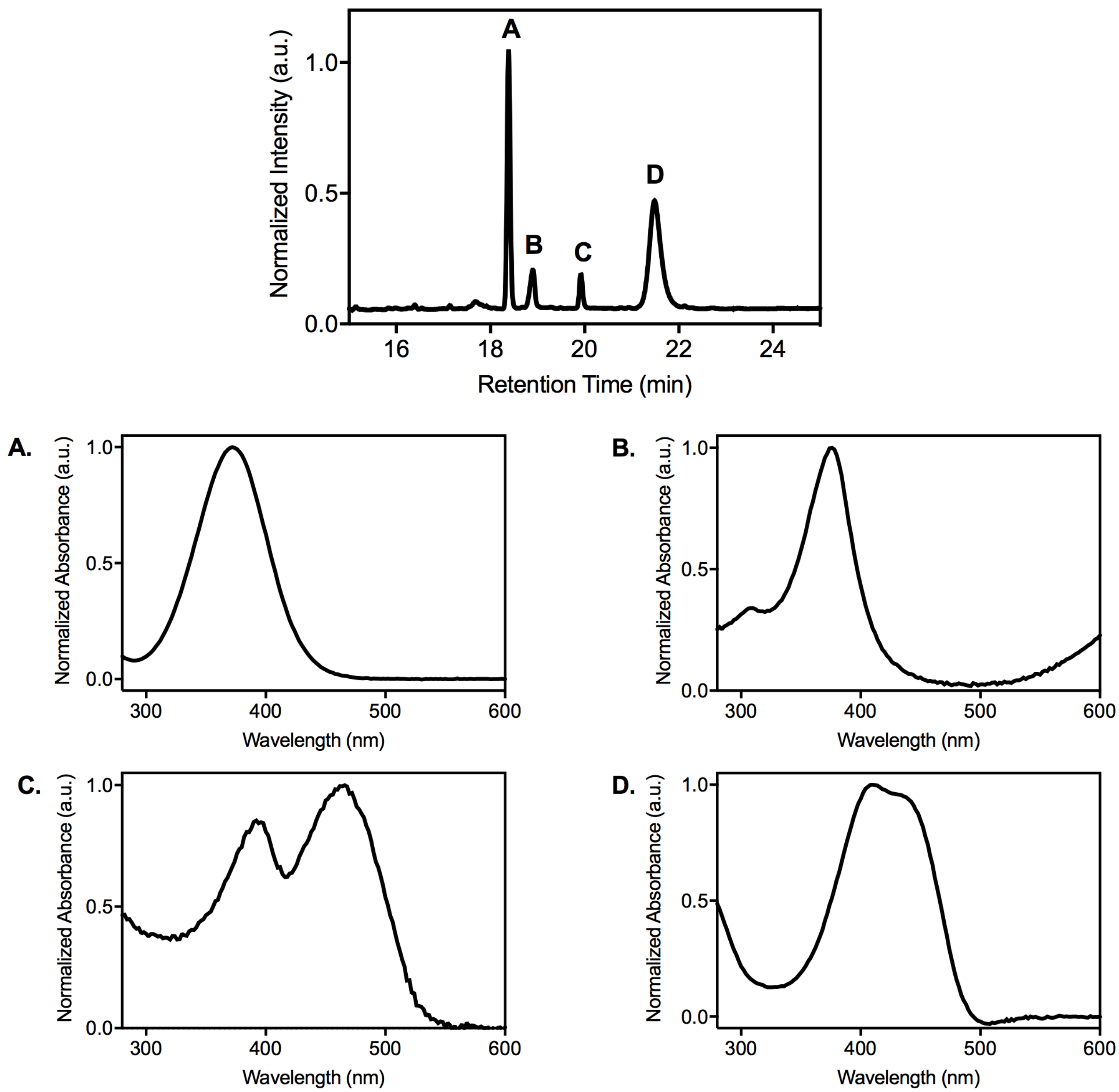

Figure S19. RP-HPLC Chromatogram at 400 nm and (bottom) the UV-vis spectra of the fractions indicted in the chromatogram: A: unknown light yellow pigment with low resolution mass of 282.1 m/z (cf. Figure S20); B: biliverdin (cf. Figure S21); C: unknown pigment with low resolution mass of 287.1 m/z (cf. Figure S22); D: bilirubin (cf. Figure S23).

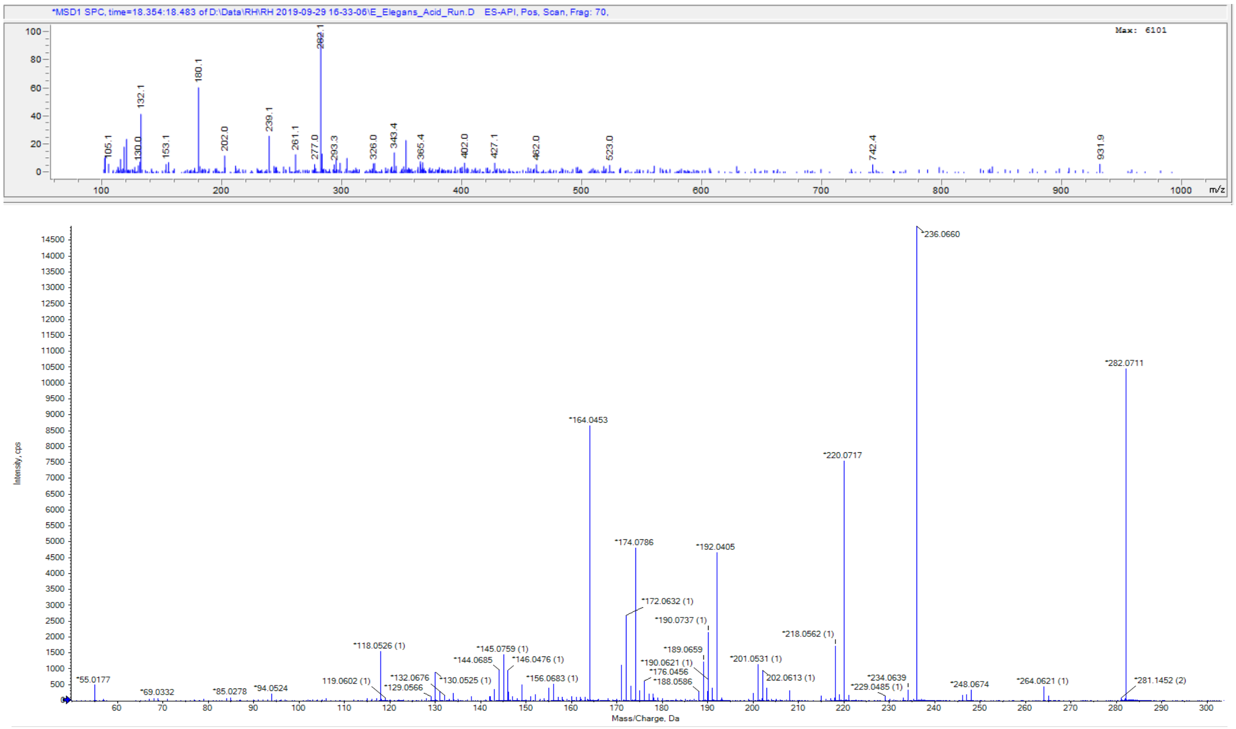

Figure S20. (top) ESI+ Low resolution LC-MS spectrum of peak A extracted *E. elegans* eggshells using the EDTA extraction protocol (cf. Figure S19A) and (bottom) from RP-HPLC HR ESI+-MS^2 spectrum (QTOF, 100% MeOH) (100% MeOH) of peak A. A structure/composition could not be assigned.

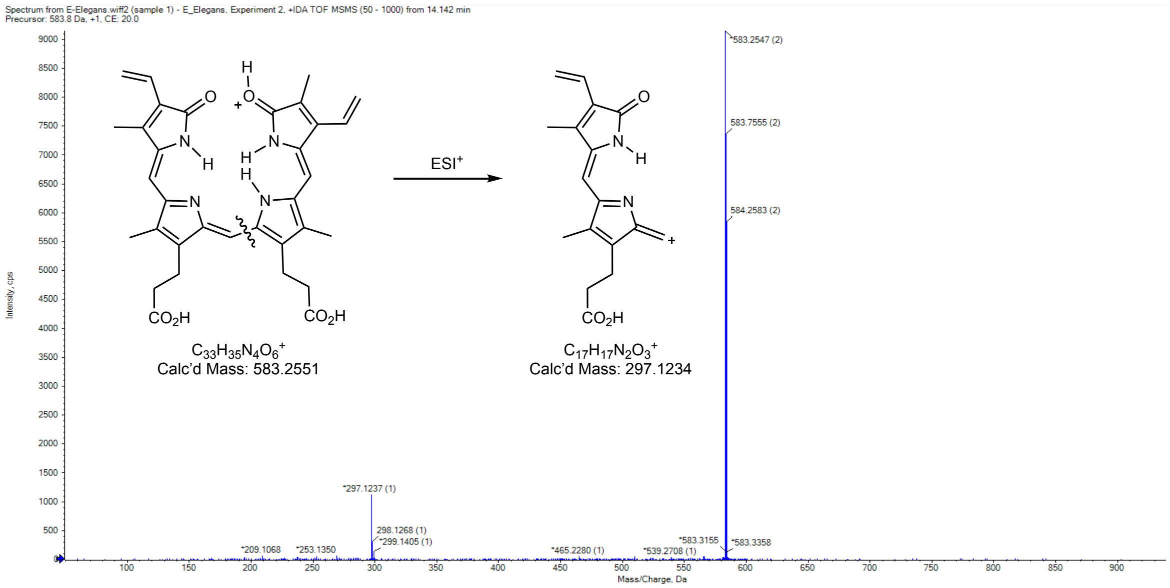

Figure S21. RP-HPLC HR ESI+-MS^2 spectrum (QTOF, 100% MeOH) of **2^H** extracted from *E. elegans* eggshells using the EDTA extraction protocol. For comparison to a commercial sample of **2^H**, cf. Figure S8.

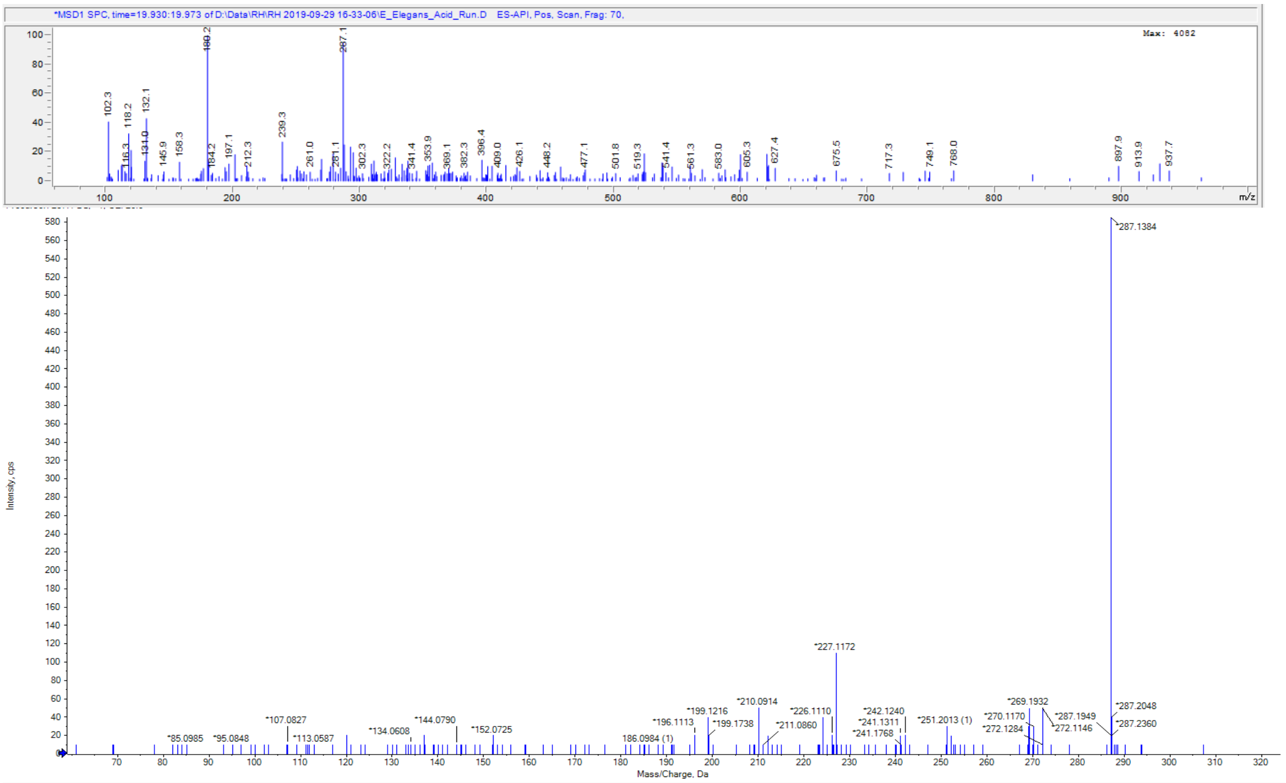

Figure S22. (top) ESI+ Low resolution LC-MS spectrum of peak C extracted *E. elegans* eggshells using the EDTA extraction protocol (cf. Figure S19C) and (bottom) RP-HPLC HR ESI+-MS^2 spectrum (QTOF, 100% MeOH) of peak C. A structure could not be assigned.

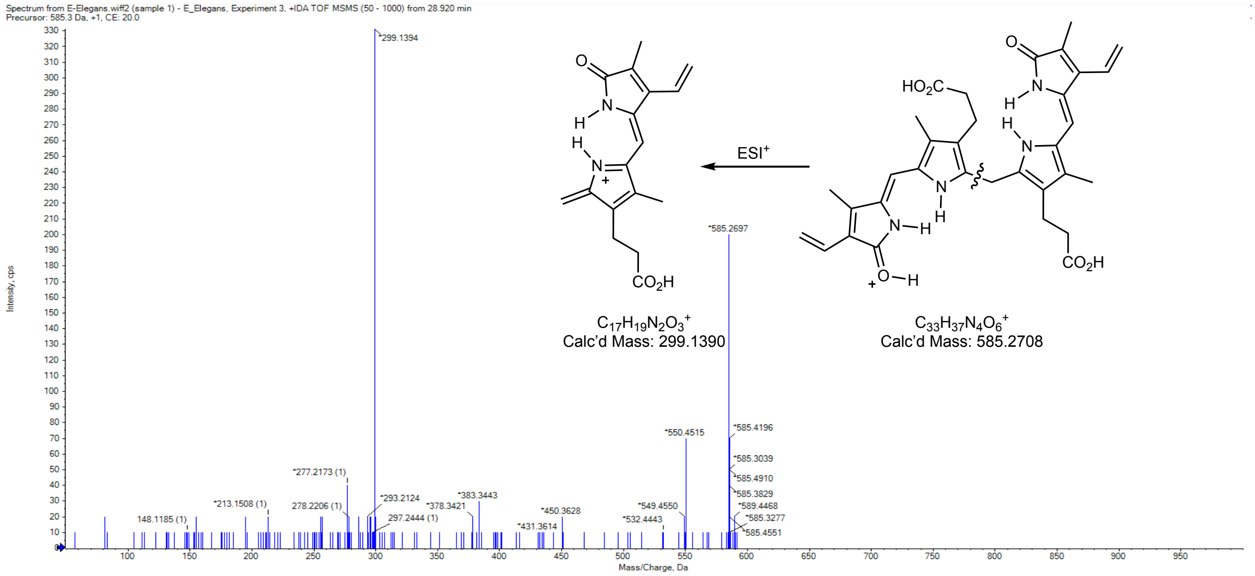

Figure S23. RP-HPLC HR ESI+-MS^2 spectrum (QTOF, 100% MeOH) of bilirubin **4^H** extracted from *E. elegans* eggshells using the EDTA extraction protocol shown in Figure S19 (Fraction D). For comparison to a commercial sample of **4^H**, see Figure S10.

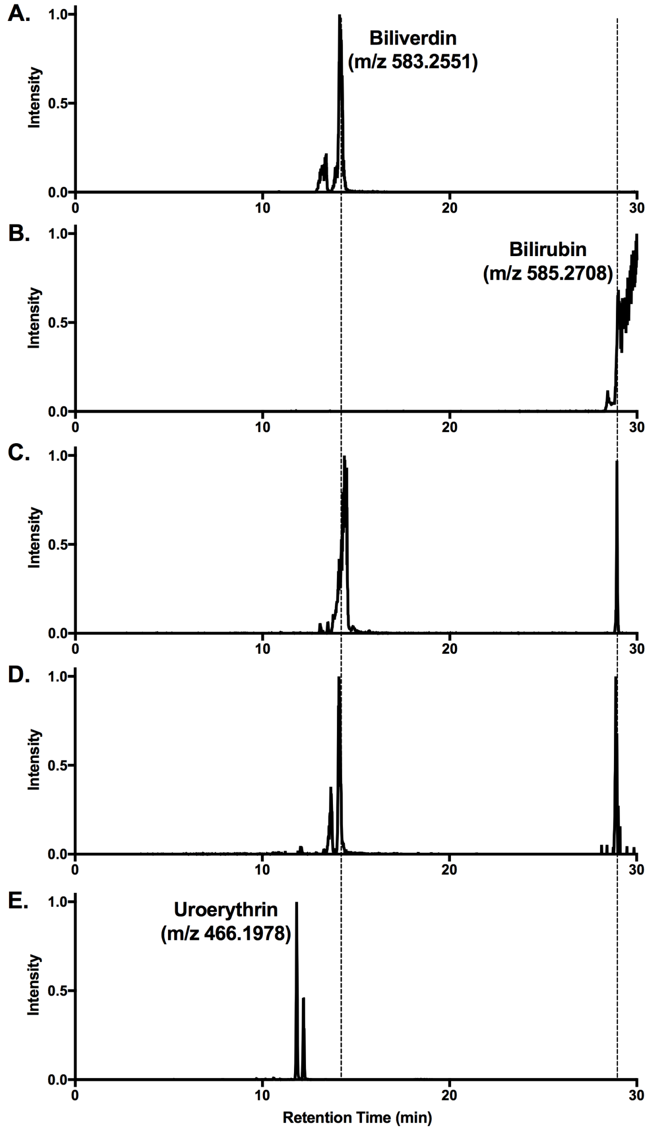

Figure S24. ESI+ extracted ion chromatograms (EICs) of LC-MS traces for crude EDTA eggshell extracts, biliverdin diacid standard, and bilirubin diacid standard. **A.** Normalized 583.2 *m/z* EIC of biliverdin diacid $\mathbf{2^H}$ standard. **B.** Normalized 585.2 *m/z* EIC of bilirubin diacid $\mathbf{4^H}$ standard. **C.** Normalized 585.2 *m/z* EIC of crude *N. maculosa* eggshell extract, showing only the bilirubin diacid $\mathbf{4^H}$ fraction. **D.** Normalized 585.2 EIC of crude *E. elegans* eggshell extract, showing only the biliverdin diacid $\mathbf{2^H}$ fraction. The splitting of the peak for biliverdin is an indication of the presence of geometric isomers.[9] **E.** Normalized 466.2 *m/z* EIC of crude *N. maculosa* eggshell extract. The splitting of the uroerythrin peak is due to the presence of regioisomers (biotripyrrin a = uroerythrin, and biotripyrrin b), particularly when resulting from the non-enzymatic photooxidation of bilirubin or biliverdin.[11]

Colour Mixing Modelling

To determine what combination of biliverdin and novel pigment (bilirubin for *E. elegans* and uroerythrin for *N. maculosa*) could produce these novel colours, we simulated the variable admixture of the two main eggshell pigments for each species using measured transmission spectra (see above), and compared these predicted spectra to measured eggshells reflectance. We measured the spectral reflectance of preserved *E. elegans* (N = 1) and *N. maculosa* (N = 1) eggshells, and also fresh gray catbird *Dumetella carolinensis* (N = 1) eggshells, using a field portable spectrometer (Jaz, Ocean Optics) relative to a diffuse white standard (WS-1, Ocean Optics). Each eggshell was measured six times: twice at the blunt pole, equator, and sharp pole. For each species, we normalized the transmission spectra of all of their extracted pigments by dividing by the maximum value for these pigments (e.g., bilirubin had a transmission of 106.85% for *E. elegans*, while an unidentified pigment had a transmission of 98.44% in *N. maculosa*). The solvents shifted the peak transmission for biliverdin relative to the peak reflectance of the pure blue-green eggshell of *D. carolinensis* (peak at 501 nm) measured in the field by 14 nm and 21 nm (*E. elegans* and *N. maculosa*, respectively); therefore, we shifted each spectrum so that the peak transmission of biliverdin matched the field measured peak reflectance. We then mixed the two major pigments, using a simple subtractive colour mixing model:

eq. 1 $$R_{\lambda} = \prod_{i=\lambda_i}^{n} R_{i,\lambda}^{c_i}$$

where *R* represents reflectance and *c* represents the relative concentration of each colourant across all wavelengths. In these calculation we varied the concentration of each colourant to generate a range of transmission spectra representing the colours of variable mixtures (N = 1001) of the two pigments (Fig 4a, b). Because the transformation resulted in missing values in the short wavelength range (< 314 nm and 321 nm respectively), we extrapolated these data using a natural spline. This resulted in reflectance values ranging from 300 to 700 nm.

Measured and predicted spectra were then converted to coordinates within the CIE colour space (Fig 4c, d)[12]. To determine the admixture ratio (novel pigment: biliverdin) we then calculated the Euclidean distance between the coordinates within this colour space for each simulated intermediate spectrum and each measured reflectance spectrum for each species (N = 6). The admixture that resulted in the smallest Euclidean distance against any measured egg spectrum was recorded. The coordinates of these 'best-matched' predictions were used to

determine the likely variable contribution of these two pigments and are depicted on the CIE plots (on Fig. 4c and d).

We then plotted the spectral reflectance of each eggshell alongside the simulated spectra; however, since these simulated values were based on normalized data we needed to multiply measured spectral reflectance values by a constant. We used the constant that resulted in the smallest just noticeable difference (JND) between the constant-modified-reflectance and any simulated spectrum (the smaller the JND the less noticeable the colour difference). The JND values were calculated following previous research in this group,[13] using a receptor noise limited visual model[14] to compare the perceived achromatic difference between each simulated colour and the measured spectral reflectance modified by a constant (across a range of potential constants). For these visual models, we used photoreceptor sensitivities for the average ultraviolet sensitive viewer[15] and the double cone sensitivity estimates for the blue-tit (*Cyanistes caeruleus*), because tinamous are believed to be sensitive to ultraviolet light.[16,17] We assumed ideal (constant at 100%) illumination and transmission for these calculations.

References for Supporting Information

1. Kennedy, G. Y. & Vevers, H. G. Eggshell Pigments of the Araucano Fowl. *Comp. Biochem. Physiol.* **44B**, 11–25 (1973).

2. Halepas, S., Hamchand, R., Lindeyer, S. E. D. & Brückner, C. Isolation of Biliverdin IXα, as its Dimethyl Ester, from Emu Eggshells. *J. Chem. Educ.* **94**, 1533–1537 (2017).

3. Dean, M. L., Miller, T. A. & Brückner, C. Egg-Citing! Isolation of Protoporphyrin IX from Brown Eggshells and Its Detection by Optical Spectroscopy and Chemiluminescence. *J. Chem. Educ.* **88**, 788–792 (2011).

4. Taniguchi, M. & Lindsey, J. S. Database of Absorption and Fluorescence Spectra of >300 Common Compounds for Use in PhotochemCAD. *Photochem. Photobiol.* **94**, 290–327 (2018).

5. Shimoharada, K. *et al.* Urine Concentration of Biopyrrins: A New Marker for Oxidative Stress *in vivo*. *Clin. Chem.* **44**, 2554a (1998).

6. Taniguchi, M., Du, H. & Lindsey, J. S. PhotochemCAD 3: Diverse Modules for Photophysical Calculations with Access to Multiple Spectral Databases. *Photochem. Photobiol.* **94**, 277–289 (2018).

7. Roth, S. D. S., T.; Lightner, D. A. Intermolecularly Hydrogen-Bonded Dimeric Helices. Tripyrrindiones. *Tetrahedron* **63**, 11030–11039 (2007).

8. Gorchein, A., Lim, C. K. & Cassey, P. Extraction and analysis of colourful eggshell pigments using HPLC and HPLC/electrospray ionization tandem mass spectrometry. *Biomed. Chromatogr.* **23**, 602–606 (2009).

9. Franski, R., Gierczyk, B., Popenda, L., Kasperkowiak, M. & Pedzinski, T. Identification of a biliverdin geometric isomer by means of HPLC/ESI-MS and NMR spectroscopy. Differentiation of the isomers by using fragmentation "in-source". *Monatsh. Chem.* **149**, 995–1002 (2018).

10. Weesepoel, Y., Gruppen, H. & Vincken, J.-P. Preliminary UHPLC–PDA–ESI-MS screening of light-accelerated autoxidation products of the tetrapyrrole biliverdin. *Food Chem.* **173**, 624–628 (2015).

11. Yamaguchi, T., Shioji, I., Sugimoto, A., Komoda, Y. & Nakajima, H. Chemical Structure of a New Family of Bile Pigments from Human Urine. *J. Biochem.* **116**, 298–303 (1994).

12. Williams, D. L. *et al.* Beyond λ[lambda]max: Transforming Visible Spectra into 24-Bit Color Values. *J. Chem. Educ.* **84**, 1873–1877 (2007).

13. Hanley, D., Stoddard, M. C., Cassey, P. & Brennan, P. L. R. Eggshell Conspicuousness in Ground Nesting Birds: Do Conspicuous Eggshells Signal Nest Location to Conspecifics? *Avian Biol. Res.* **6**, 147–156 (2019).

14. Vorobyev, M. & Osorio, D. Receptor noise as a determinant of colour thresholds. *Proc. R. Soc. London, Ser. B* **265**, 351-358 (1998).

15. Endler, J. A. & Mielke Jr, P. W. Comparing entire colour patterns as birds see them. *Biol. J. Linnean Soc.* **86**, 405–431 (2005).

16. Wright, M. W. & Bowmaker, J. K. Retinal photoreceptors of paleognathous birds: The ostrich (*Struthio camelus*) and rhea (*Rhea americana*). *Vision Res.* **41**, 1–12, (2001).

17. Aidala, Z. *et al.* Ultraviolet visual sensitivity in three avian lineages: Paleognaths, parrots, and passerines. *J. Comp. Physiol. A* **198**, 495–510 (2012).
